# The promotion of sustainable land use planning for the enhancement of ecosystem service capacity: Based on the FLUS-INVEST-RUSLE-CASA model

**DOI:** 10.1371/journal.pone.0305400

**Published:** 2024-07-09

**Authors:** Guiyuan Li, Zhanneng Wu, Yu He, Chi Chen, Yan Long

**Affiliations:** 1 School of Civil Engineering, Architecture and Environment, Hubei University of Technology, Wuhan, China; 2 Key Laboratory of Intelligent Health Perception and Ecological Restoration of River and Lake, Ministry of Education, Hubei University of Technology, Wuhan, China; 3 Innovation Demonstration Base of Ecological Environment Geotechnical and Ecological Restoration of Rivers and Lakes, Hubei University of Technology, Wuhan Hubei University of Technology, Wuhan, China; 4 Hubei Academy of Agricultural Sciences, Wuhan, China; 5 School of Urban Design, Wuhan University, Wuhan, China; Jinan University, CHINA

## Abstract

Land Use/Land Cover (LULC) is one of the most significant human variables influencing the efficiency of Ecosystem Services (ESs) in terrestrial ecosystems. Theoretical and technical assistance for regional sustainable land use planning and management, as well as ecosystem conservation and restoration, is provided by investigating the influence of changes in the LULC pattern on the efficiency of ESs. This research focuses on the interactions between socioeconomic activities and natural ecological processes in the Three Gorges Reservoir Area (TGRA). We use LULC data from the TGRA for the years 1990, 2000, 2010, and 2020. The study includes the analysis and calculation of the spatiotemporal evolution features of the current LULC pattern and the efficiency of ESs, including their spatiotemporal distribution. Considering the TGRA’s national development orientation and guidance, three potential LULC patterns are constructed under various develop-ment scenarios. To calculate the efficiency of ESs, the GeoSOS-FLUS future LULC simulation model is linked, and several methodologies such as INVEST, RUSLE, and CASA are used. The goal is to investigate the influence of future changes in LULC patterns on ESs efficiency. The findings show the following: (1) From 1990 to 2020, the values of water conservation services in the TGRA decreased and subsequently increased. High-value areas are primarily located in the reservoir’s centre and eastern sections, whereas low-value areas are mostly found in the western section. Soil conservation service values initially declined and later climbed. The TGRA’s carbon storage services have in-creased yearly, from 552.64 g/m^2^ in 2000 to 615.92 g/m^2^ in 2020. (2) In the ecological protection scenario, carbon storage and soil erosion increased compared to the ecosystem services in 2020. The ecological system service benefits are greater when compared to the natural development scenario. (3) The four ESs show positive spatial correlations across all three scenarios, and local spatial au-tocorrelation analysis findings demonstrate that carbon storage, water yield, and habitat quality have comparable spatial distributions across all three scenarios. To some extent, high-value areas for water conservation, soil retention, carbon storage, and habitat quality overlap.

## 1. Introduction

"Land Use/Land Cover (LULC)" acts as a link between human social activities and the interaction with terrestrial Ecosystem Services (ESs). The human "extensive and high-speed" socioeconomic growth model and the "heavy and light" urbanization building model have resulted in constant modifications in the LULC pattern throughout the years. While humans have benefited from ESs in various ways, changes in LULC patterns have generated enormous disruptions and stresses on ESs, leading to a cascade of devastating effects. Extreme climate change [1, 2], food security [3], declining biodiversity [4, 5], soil retention [6, 7], and increased carbon emissions [8, 9] all represent important problems. The increase in human activities, according to the World Wide Fund for Nature’s "Living Planet Report 2022" [10], poses a significant danger to the Earth’s Ecosystem Structure-Function-Services capability. In response to these difficulties, the United Nations Environment Program has launched a range of initiatives to promote environmental preservation and foster sustainable development that is in harmony with nature. Initiatives such as the "Decade on Ecosystem Restoration (2021–2030)" expressly highlight the challenges presented to the entire natural world by climatic emergency, natural deterioration, and lethal pollution, with up to a million plant and animal species risking extinction [11]. In the report titled "2022 edition of the Emissions Gap Report: The Closing Window," it is noted that: "Existing climate commitments would lead to a global temperature rise of 2.4–2.6°C by the end of this century. To limit global warming to within 1.5°C, urgent progress is needed in the comprehensive transformation of the entire system, along with robust financing for implementation and adaptation actions." The Food and Agriculture Organization’s (FAO) "2022 State of the World’s Forests" study stated: "Human socio-economic activities lead to an annual depletion of global forest resources, causing approximately 10% of the total global ecosystem production loss, and thereby undermining the well-being of 3.2 billion people worldwide" [12]. The continually shifting LULC pattern caused by human socioeconomic activities has impacted the efficiency of Earth’s ESs, posing a serious threat to human safety, health, and well-being. As a result, one of the most important parts of study in the field of sustainable development is determining future trends in LULC pattern changes and their influence on ESs efficiency.

Human research of ESs began with an emphasis on the biological and ecological interaction between the biological components and the inorganic environment [13]. Researchers have increasingly understood that the services ecosystems supply to people contain both good and negative elements as they have investigated ESs’s components, structures, processes, and functions. Following that, specialists and academics from numerous domains such as ecology, geography, environmental science, economics, and urban studies have performed substantial studies on the influence of human socioeconomic activities on ESs from a socio-ecosystem viewpoint. This study is being undertaken with a socio-ecosystem approach in mind. Ehrlich defined and outlined the idea and features of "ecosystem services" [14] (Ehrlich, 1983), launching worldwide qualitative research on the concept and substance of ESs [15]. Robert Costanza and Gretchen Cara Daily published two key research contributions in 1997 [16, 17], broadening the study of ESs to incorporate categorization systems and quantitative assessments [18–21]. The United Nations Millennium Ecosystem Assessment [22] program defined the categorization of ESs, which includes supplying, sustaining, regulating, and cultural services. This program redirected the focus of ESs research toward understanding processes, trade-offs, and synergies [23–25], as well as their connections to human well-being and sustainable development.

The investigation of the link between LULC pattern changes and terrestrial ESs has become a hot issue since it is regarded as one of the most important anthropogenic driving forces impacting terrestrial ESs. International scholars have researched the impact of LULC changes on ESs in a variety of climatic zones and spatial regions, including arid regions [26], semi-arid agro-pastoral transitional zones [27, 28], aquatic-terrestrial transition zones [29], watershed areas [30, 31], coastal regions [32, 33], and metropolitan areas [34, 35]. These studies investigate the value, spatiotemporal changes, supply-demand linkages, trade-offs, and synergies of ESs impacted by LULC pattern changes on a single and multi-factor basis. Fisheries conservation, carbon storage, water quality, soil retention, food security, and other topics are covered [13, 36, 37]. Remote sensing (RS), GIS spatial analysis tools, and the examination of various forms of land conversion are frequently used in research approaches to assess changes in LULC patterns. Models such as ARIES, SoLVES, and InVEST are also used to investigate the effects of LULC changes on the value, regional heterogeneity, spatiotemporal evolution processes, and other aspects of ecosystem services (ESs) [38–40]. In addition, mathematical and analytical tools are used to investigate the trade-offs and synergies in the influence of LULC modifications on ESs [41, 42].

Predicting the impact of future dynamic changes in LULC patterns on ESs has become a hot topic in fields such as ecology, land management, environmental science, and territorial spatial planning, thanks to advances in research and the integration of spatial information technology, big data analytics, and the application of multisource remote sensing data. Cellular Automaton (CA) [43], Markov models, CLUE-S [44], FLUS [45], Plus [46], and other models are now utilized to simulate and forecast the dynamic changes in future LULC patterns. Experts and researchers using LULC models in practical research have discovered flaws in each simulation model. As a result, better models such as Markov-CA, CA-CLUS-S, and others have been used in research to overcome these flaws. FLUS is an upgraded model based on Geo-SOS that integrates Cellular Automaton (CA) and Artificial Neural Networks (ANN) functioning concepts. It employs an adaptive inertia coefficient and a roulette competition mechanism to simplify the overall influence of natural and social elements. FLUS quantifies the states and rates of land use type transitions, improving land use modeling accuracy. It is now a crucial approach in LULC modeling and prediction [47].

In summary, academics have mainly concentrated on investigating the influence of LULC pattern changes on ESs, emphasizing research in temperate zones, regions with typical features, and big metropolitan areas. There has been relatively little research into basin reservoir areas generated by hydroelectric developments. There is still a scarcity of studies on accurately forecasting the future trends of LULC pattern changes and exploring the spatiotemporal features, driving variables, and processes of the overall impacts on ESs in these places. The Three Gorges Reservoir Area (TGRA) is a distinct geographical area developed due to the Three Gorges Water Control Project. It is an important ecological protection region in the Yangtze River Basin, an important ecological functional area in the Yangtze Economic Belt, and a significant hub for the Chengdu-Chongqing Urban Agglomeration as well as the urban agglomeration in the Yangtze River’s middle and upper reaches. Coordinating high-level protection and high-quality development in this region has become a significant priority for government officials and specialists. Based on the TGRA’s distinct geographical position, biological environment, and socioeconomic development state, this study examines the considerable changes in its LULC patterns due to large-scale hydropower projects and urbanization. It also investigates the current state of the reservoir’s ecosystem services efficiency. The project attempts to forecast future LULC pattern changes in the TGRA with better precision using the combined GeoSOS-FLUS model. Furthermore, the study extensively examines the overall factor service efficiency of ecosystem services by combining models such as InVEST, NDVI, RUSLE, CASA, and others. The goal is to determine the influence of future LULC pattern changes on the efficiency of ecosystem services and to investigate future sustainable LULC planning patterns based on improving ecosystem services efficiency in the watershed reservoir region. This research provides technical analysis methodologies and data support for the TGRA’s long-term LULC planning and administration. It intends to contribute to the Yangtze River Basin’s high-level governance and high-quality development.

ESs refers to all of the advantages that humans derive directly or indirectly from ecosystems, including physical, ecological products, and intangible services. [48]. Costanza [49] et al. classified ecosystems into 17 primary groups based on the renewable services they provide. According to the United Nations Millennium Ecosystem Assessment report, ecosystem services are classified into four categories: Supporting services comprise functions that sustain the nutrient cycles required for Earth’s survival environment; Provisioning services are functions that offer basic necessities such as food, water, and air; Regulating services are functions that govern and regulate environmental balance and stability, such as flood control, climate control, and carbon sequestration; Cultural services are intangible advantages relating to human well-being, enjoyment, and cultural values that represent cultural links between humans and the environment. This categorization system aids in the thorough knowledge and appraisal of ecosystems’ varied contributions to human society.

## 2. Study area and data

### 2.1. Study area

**Fig 1:**
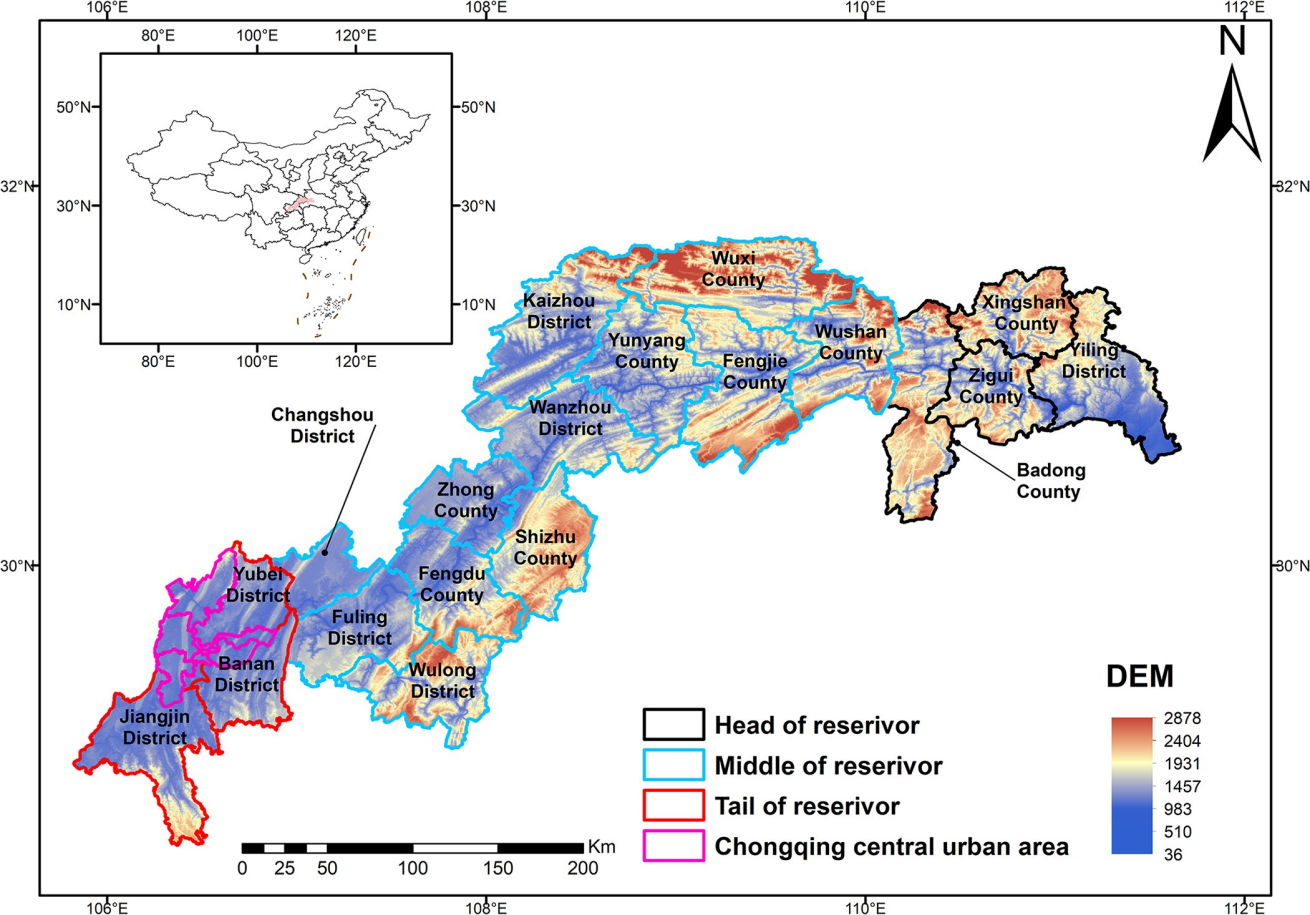
Location map of the Three Gorges Reservoir Area. This is a map of the geographic location of the Three Gorges Reservoir Area, including the administrative area of the Three Gorges Reservoir Area, altitude.

The TGRA(28°52′–31°75′N, 105°82′–111°66′E) is a unique geographical entity that arose due to the development of the Three Gorges Water Control Project [50]. Its territorial space comprises the submerged territory at the Three Gorges Dam’s typical water level of 175 meters and the people relocation area. This area includes 30 districts (counties) in the Hubei and Chongqing sections, totalling 56,700 square kilometres. The Chongqing section covers 79% of the reservoir area, with 22 districts (counties), while the Hubei section covers 21% of the reservoir area, with 8 districts (counties) (Fig 1) [51].

The TGRA is located at the crossroads of three important tectonic units: the Daba Mountains, the eastern half of Sichuan, and the Chuan-E Yu-Huai fold belt. The geography is dominated by mountains and hills. The LULC pattern indicates that there is a significant human-environment conflict inside the reservoir region. On the one hand, cultivated land is scarce per capita, with a predominance of arid slope land, which has resulted in substantial land development. However, there is a growing tension between agricultural and non-agricultural land usage. The bulk of farmland inside the reservoir region is of poor quality, demonstrating a clear contradiction between a lack of farmland and low grain production. From the standpoint of ESs: During the Three Gorges Project’s construction, socioeconomic activities, and urbanization expansion inside the reservoir region caused additional imbalances in the LULC pattern, significantly reducing the capacity of ecosystem services. The LULC pattern inside the reservoir region has improved with the adoption of succeeding plans, such as the post-Three Gorges planning and Yangtze River conservation programs. To some extent, this enhancement has helped to increase the efficacy of ecosystem services in the reservoir region.

### 2.2. Data socure

#### 2.2.1. Ecosystem services calculation-related data

In this study, the TGRA is selected as the research unit, and the data utilized include: Land Use Data for the years 1990, 2000, 2010, and 2020 from Landsat (http://landsat.visibleearth.nasa.gov/). The spatial resolution is 30m × 30m.DEM data obtained from the Geospatial Data Cloud (https://www.gscloud.cn/) with a spatial resolution of 30m.

Meteorological data from the National Earth System Science Data Center (https://www.geodata.cn/) for the years 1990, 2000, 2010, and 2020, including temperature and precipitation data. The spatial resolution is one kilometre. Soil data taken from the World Soil Data (HWSD) China Soil Characteristics Dataset on the Geography Data Platform of Peking University’s College of Urban and Environmental Sciences (http://geodata.pku.edu.cn/). The spatial resolution is one kilometre. Statistical Yearbooks for Chongqing Municipality and Hubei Province for 1990, 2000, 2010, and 2020.

#### 2.2.2. FLUS model-related land use and driving factor data

Landsat http://landsat.visibleearth.nasa.gov) provided the land use type data. Data for four periods of land use remote sensing monitoring (1990, 2000, 2010, and 2020) were gathered, with a spatial resolution of 30m. Theland was classified into three levels, and a two-level classification system was chosen for the actual research, dividing the study area into nine land types: paddy fields, dryland, forest, grassland, water area, urban land, rural residential areas, industrial land, and unused land. The first-level classification system was used in the study of current land-use change(1990–2020): farmland, forest, grassland, water area, construction land, unused land. In this study, the driving factors for land use change were population, DEM values, soil type, yearly average rainfall, annual average temperature, distance to railroads, distance to national highways, GDP, and distance to county roads. Landsat (http://landsat.visibleearth.nasa.gov/) also provided statistics on DEM values, population, distance to railroads, distance to national highways, GDP, and distance to county roads. The National Meteorological Information Center (https://data.cma.cn/) provided yearly average rainfall and temperature data. Table 1 shows the summary statistics for each kind.

**Table 1:** Remote sensing datasets of land use and driving factors.

Categories	Factors	Resolution	Data Sources	Time (Year)
Land use data	Land use remote sensing monitoring data(Landsat-TM/ETM)	30 m	http://landsat.visibleearth.nasa.gov/	1990 2000, 2010, 2020
Natural geographic data	DEM	30 m	SRTM V3	2015
	Annual average temperature	1 km	http://landsat.visibleearth.nasa.gov/	1990 2000, 2010, 2020
	Annual average precipitation	1 km	http://landsat.visibleearth.nasa.gov/	1990 2000, 2010, 2020
	NDVI	1 km	http://landsat.visibleearth.nasa.gov/	1990 2000, 2010, 2020
	Soil type	1 km	http://geodata.pku.edu.cn/	1990
Socioeconomic data	Chongqing Statistical Yearbook	/	https://www.cq.gov.cn/	1990 2000, 2010, 2020
	Hubei Statistical Yearbook	/	https://www.hubei.gov.cn/	1990 2000, 2010, 2020
	Population	1 km	http://landsat.visibleearth.nasa.gov/	2020
	Distance from the railway	1 km	http://landsat.visibleearth.nasa.gov/	2020
	Distance from the national highway	1 km	http://landsat.visibleearth.nasa.gov/	2020
	GDP	1 km	http://landsat.visibleearth.nasa.gov/	2020

## 3. Methodology

### 3.1. Research process

**Fig 2:**
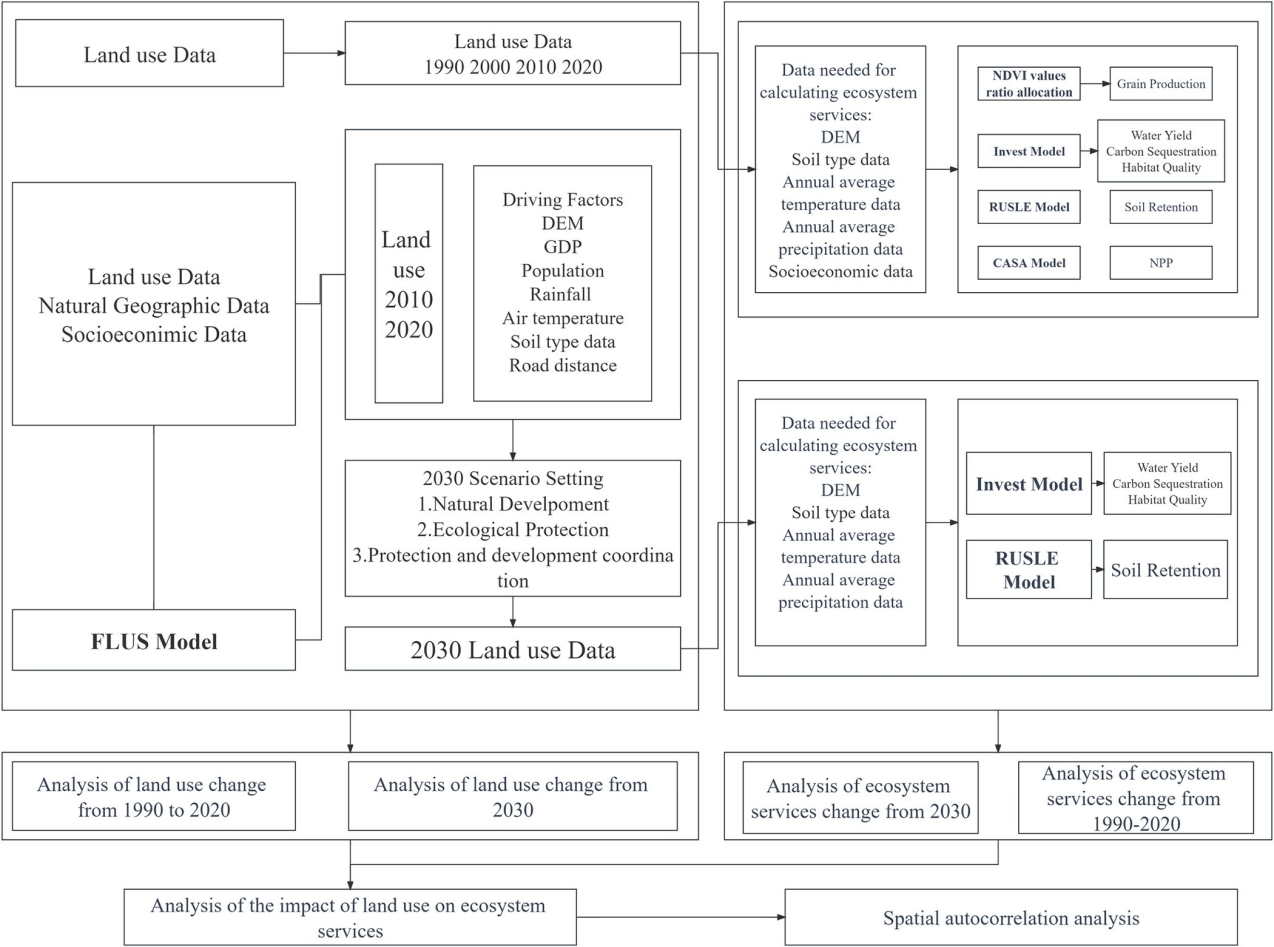
Technical flow chart. This figure illustrates the research flow of the article.

This study selects six types of ecosystem services from three major categories: supporting services, provisioning services, and regulating services. (grain production, water yield, carbon sequestration,soil retention, habitat quality and net primary production)The InVEST model, CASA model, and RUSLE model are utilized to assess the current status of ecosystem services in the Three Gorges Reservoir Area. Combined with future land use and land cover (LULC) trend simulations, predictions for future ecosystem services are made. This approach aims to explore sustainable planning strategies for regional land use and land cover, providing a theoretical basis for the development of ecological civilization in the Three Gorges Reservoir Area. The technical roadmap is illustrated in Fig 2.

### 3.2. Land use change predictionbased on the FLUS model

The FLUS model was created to simulate land use changes caused by human activities and natural forces, as well as to estimate future land use scenarios [52]. The FLUS model incorporates an adaptive competition mechanism that successfully tackles the unpredictability and complexity involved with the mutual change of multiple land use types as a result of the combined impact of natural processes and human activities. Compared to other land use simulation models such as CA-Markov PLUS, the FLUS approach is more advanced and is capable of making predictions with longer time dimensions. This feature gives the FLUS model exceptional simulation accuracy, allowing it to deliver results that are very near to real-world land use patterns.

This study applies the FLUS2.4 model, with 2010 land use data as a baseline, to forecast the ecological changes in the TGRA properly in 2030. Simultaneously, we chose nine driving elements that drive land use change, taking into account natural geographical characteristics, climatic conditions, and socioeconomic backgrounds. We guarantee that the model can effectively reflect real-world events in simulations and increase its accuracy in projecting ecological changes by 2030 by modifying the transfer matrix parameters and neighbourhood factor parameters numerous times and adding a land simulation conversion matrix for the different scenarios. (Table 2) In the transfer matrix, 0 implies that no conversion is feasible, whereas 1 indicates that conversion is possible. The neighbourhood factor weight varies from 0 to 1, with values closer to 1 suggesting a land class’s capacity to expand." "The TGRA’s land use shift in 2020 was simulated and compared to real land use type data for the same year.

**Table 2:** Cost matrix for land conversion simulation under different scenarios in the year 2030.

	Natural Development Scenario									Ecological Protection Scenario									Protection and development coordination scenarios								
	P	D	F	G	W	U	R	I	UL	P	D	F	G	W	U	R	I	UL	P	D	F	G	W	U	R	I	UL
P	1	1	1	1	1	1	1	1	0	1	1	1	1	1	0	0	0	0	1	1	1	1	0	0	1	0	0
D	1	1	1	1	1	1	1	1	0	1	1	1	1	1	0	0	0	0	1	1	1	1	1	0	1	0	0
F	1	1	1	1	1	1	1	1	0	0	0	1	1	1	0	0	0	0	0	0	1	1	0	0	0	0	0
G	1	1	1	1	1	1	1	1	0	0	0	1	1	1	0	0	0	0	0	0	1	1	0	0	0	0	0
W	1	1	1	1	1	0	1	0	0	0	0	1	1	1	0	0	0	0	0	0	1	1	1	0	0	0	0
U	0	0	0	0	0	1	0	0	0	0	0	0	0	0	1	0	0	0	1	1	0	0	0	1	1	1	0
R	1	1	1	1	1	0	1	0	1	1	1	1	1	1	0	1	0	0	1	1	0	0	0	1	1	1	0
I	0	0	0	0	0	0	0	1	0	0	0	0	0	0	0	1	0	0	0	0	0	0	0	1	1	1	0
UL	1	1	1	1	1	1	1	1	1	1	1	1	1	1	1	1	1	1	1	1	1	1	1	1	1	1	1

Note: “1” means conversion is allowed, and “0” means conversion is not allowed. P stands for paddy fields, D for dryland, F for forestland, G for grassland, W for water area, U for urban land, R for rural settlements, I for Industrial and mining land, UL for unused land.

In order to confirm the accuracy of the classification, the kappa standard, kappa location, kappa no. and FoM in the FLUS module were calculated. FoM is a number between 0 and 1, indicating complete overlap (1) and no overlap (0) between the simulated and real maps, respectively. FoM was obtained using Eq (1) [53].


$$FoM=\frac{Hits}{Misses+Hits+False\;Alarms}$$ (1)

(1) where Hits denotes the correct pixels where land use change has occurred in the observed and simulated data; Misses means the pixels that were fixed in the simulated data, although in the observed data they have changed; and False Alarms are errors that the model predicts changed but did not do so in observation.

The correctness of the simulation results was confirmed using overall accuracy, kappa coefficient, and Figure of Merit (FoM) values. The simulation findings’ overall accuracy and kappa were 90.16% and 81.39%, respectively, showing strong simulation performance and high credibility, with a high level of consistency in the projected outcomes. Pontius said that the FoM index outperforms the kappa coefficient in determining the correctness of simulated changes. The FoM in this investigation was 0.02, which met the research criteria."

### 3.3. Setting scenarios for FLUS model development

The scenario for this study is based on the TGRA’s substantial conflicts between humans and the environment, the ecological environment’s fragility, severe soil erosion, and regular geological disasters. Simultaneously, it takes into consideration national development plans, particularly advice for the TGRA’s sustainable growth, such as the Yangtze River’s grand protection and the Yangtze River Economic Belt’s high-quality development, among other key national policies. In this context, we have defined the TGRA’s substantial importance as an important ecological protection zone in the Yangtze River basin and an important ecological functional region in the Yangtze River Economic Belt. The coordinated progress of high-level protection and high-quality development in the TGRA has become an effective way of executing national strategies within this framework. Referring to the X Liu study [45] and the generalized parameter settings of the model, this study presents three potential LULC pattern development possibilities and sets the land simulation weights under different models Table 3. (1) Natural Development Scenario: Various planning rules and legal laws governing land use changes are ignored. The simulation mimics the natural evolution patterns of land use structures, allowing for reciprocal conversions between different types of land. The only limitations apply to the conversion of forest land, building land, and unused land into water areas. The forecast of predicted land use changes in 2030 is based on the transition rates of land use from 1980 to 2020. (2) Ecological Protection Scenario: Adherence to present ecological protection policies is stressed in this scenario, with an emphasis on ecological planning to prevent inappropriate occupation of diverse land resources. The TGRA’s forest and agriculture will be successfully safeguarded. Conversion of forest land, agricultural, and water areas into development land will be less likely. As limited conversion zones, defined ecological redlines, nature reserves, and the limits of permanent basic farming and urban development will be considered. (3) Protection and development coordination scenarios: There are no constraints on the conversion of various land types into building land in this scenario, allowing for mutual conversions between urban and rural regions. The focus, however, is on the preservation and development of ecological land.

**Table 3:** Domain weights for land simulation under different scenarios in the year 2030.

Scenario types	P	D	F	G	W	U	R	I	UL
Natural Development Scenario	0.3	0.3	0.4	0.4	0.5	0.8	0.6	1	0.8
Ecological Protection Scenario	0.3	0.3	1	1	1	0.6	0.5	0.8	0.7
Protection and development coordination scenarios	0.3	0.3	0.5	0.5	0.5	1	1	0.8	0.7

### 3.4. Ecosystem services assessment

Three categories and six types of ESs were chosen for the study based on the features of the study region as well as the present state and hotspots of ESs research. The provisioning services are grain production (GP) and water yield (WP), the regulating services are carbon sequestration (CS) and soil retention (SR), and the supporting services are habitat quality (HQ) and net primary production (NPP). Table 4 describes the methodologies for determining ecosystem services.

**Table 4:** Measurement methods for ecosystem services.

Types	Methods	Formulas	Significance of Metrics
Grain Production	Allocate the total grain production based on the ratio of the grid NDVI values to the regional total NDVI values [54]	$G_{\left(x\right)}=\frac{{NDVI}_{x}}{{NDVI}_{sum}}\times G_{sum}$ (2)	*G*_(*x*)_ is the grain production; *NDVI*_*x*_ is the NDVI value of raster; *NDVI*_*sum*_ is the sum of NDVI values of the region; *G*_*sum*_ is the total grain production
Water Yield	InVEST model Water Yield module [55]	$Y_{\left(x\right)}=\left(1-\frac{{AET}_{\left(x\right)}}{P_{\left(x\right)}}\right)\times P_{\left(x\right)}$ (3)	*Y*_(*x*)_ is water yield (mm); *AET*_(*x*)_ is annual actual evapotranspiration (mm); *P*_(*x*)_ is annual precipitation (mm)
Carbon Sequestration	InVEST Model Carbon Sequestration Module [56]	$C_{total}=C_{above}+C_{below}+C_{soil}+C_{dead}$ (4)	*C*_total_ is total carbon (t); *C*_*above*_、*C*_*below*_、*C*_*soil*_、*C*_*dead*_ are above-ground, below-ground, soil, and dead biogenic carbon stocks, respectively
Soil Retention	RUSLE model [57]	*A = RKLSCP* (5)	*A* is the soil retention modulus; *R* is the rainfall erosivity factor; *K* is the soil erodibility factor; *LS* is the slope length and gradient factor; *C* is the vegetation cover factor; *and P* is the soil conservation measure factor
Habitat Quality	InVEST Model Habitat Quality Module [58]	$Q_{xj}=H_{xj}\left[1-(\frac{D_{xj}^{z}}{D_{xj}^{z}+k^{z}})\right]$ (6)	*Q*_*xj*_ is the habitat quality index; *H*_*xj*_ is habitat suitability; *D*_*xj*_ is the degree of habitat degradation; *k* is the saturation coefficient; *z* is a constant
Net primary production	CASA Model [59]	*NPP*(*x*,*t*) *= APAR*(*x*,*t*)*×ε*(*x*,*t*) *ε*(*x*,*t*) *= T*(*x*,*t*)*×W*(*x*,*t*)*×ε*_*max*_ (7)	*NPP*(*x*,*t*) is net first productivity; *APAR*(*x*,*t*) is light and effective radiation; *ε*(*x*,*t*) is actual light energy utilization; *ε*_*max*_ is the maximum light energy conversion rate under ideal conditions; *T*(*x*,*t*) is the temperature stress coefficient; *W*(*x*,*t*) is the water stress coefficient

### 3.5. Spatial autocorrelation analysis

Spatial autocorrelation analysis can be used to determine if the distribution of geographical data is clustered. Both global and local spatial autocorrelation can efficiently explain the interactions between geographical entities, estimating the degree of aggregation or dispersion among phenomena’ spatial properties [60]. Spatial autocorrelation analysis includes global spatial autocorrelation [61] and local spatial autocorrelation [62].

#### 3.5.1. Global spatial autocorrelation


$$MI=\frac{\sum_{i=1}^{n}\sum_{i=1}^{n}w_{ij}(x_{i}-\bar{x})(x_{j}-\bar{x})}{S^{2}\sum_{i=1}^{n}\sum_{j=1}^{n}w_{ij}}$$ (8)

The *MI* values in Formula (8) vary from 0 to 1. A positive number indicates that the geographical distribution of the geographic entity’s attribute values displays positive spatial autocorrelation. The closer the value is to 1, the closer the units are connected. A negative value implies that the property values’ geographic distribution has negative spatial autocorrelation. A value of 0 indicates that there is no spatial autocorrelation in the geographical distribution of the geographic entity’s attribute values.

#### 3.5.2. Local spatial autocorrelation

This study divides the TGRA into grids of 2000m×2000m and employs Local Indicators of Spatial Association (LISA) to depict the clustering patterns of ecosystem services in local grid spaces.


$${MI}_{i}=\frac{\left(X_{i}-\bar{x}\right)}{S^{2}}\sum_{i}w_{ij}(x_{j}-\bar{x})$$ (9)

In Eq (9),*MI*_*i*_∈[-1,1], A positive value shows geographic clustering of comparable values around the unit. The adjacent values are also high (high-high) in locations with high observed values, whereas they are low (low-low) in areas with low observed values. A negative value denotes geographical clustering of dissimilar values, with low-value areas surrounded by high values (low-high) or high-value areas surrounded by low values (high-low). A score of zero implies that there is no geographical association with nearby regions.

## 4. Result

### 4.1. Changes in land use patterns in the TGRA from 1990 to 2020

Farmland, forest land, and grassland dominate the land use types in the research region. These three land types cover 96.48% of the area along the Yangtze River in the Three Gorges Reservoir Region. The biggest share of them is forest land. In terms of regional distribution, the Hubei part of the region’s forest land area exceeded 195.69×10^8^ m^2^, accounting for more than 45% of the study area in 2020. The agriculture area in the Chongqing section accounts for more than 88% of the research area. The distribution of land use in the two administrative areas is typified by farmland dominating the Chongqing section and forest land dominating the Hubei section.

The transformation of construction land is the most important shift in the research region in terms of the degree of LULC pattern modification (Table 5). The area of building land in 1990 was 4.08×10^8^m^2^, but by 2020, it had grown to 23.86×10^8^m^2^. Farmland declined by 14.1×10^8^ m^2^, grassland decreased by 19.08×10^8^ m^2^, unused land decreased by 106.11 hm^2^, forest land increased by 8.72×10^8^ m^2^, and water area grew by 4.5×10^8^ m^2^ throughout the 30 years.

**Table 5:** Land area of various types in the TGRA from 1990 to 2020 (×10^8^ m^^2^^).

Time(year)	Land use Types					
	Farmland	Forest	Grassland	Water area	Construction land	Unused land
1990	297.53	362.94	98.73	10.30	4.08	0.1310
2000	295.46	361.63	99.75	10.37	6.37	0.1312
2010	216.23	276.21	62.24	10.88	9.72	0.0532
2020	283.43	371.66	79.65	14.80	23.86	0.1915

### 4.2. Analysis of ESs efficiency in the TGRA from 1990 to 2020

**Fig 3:**
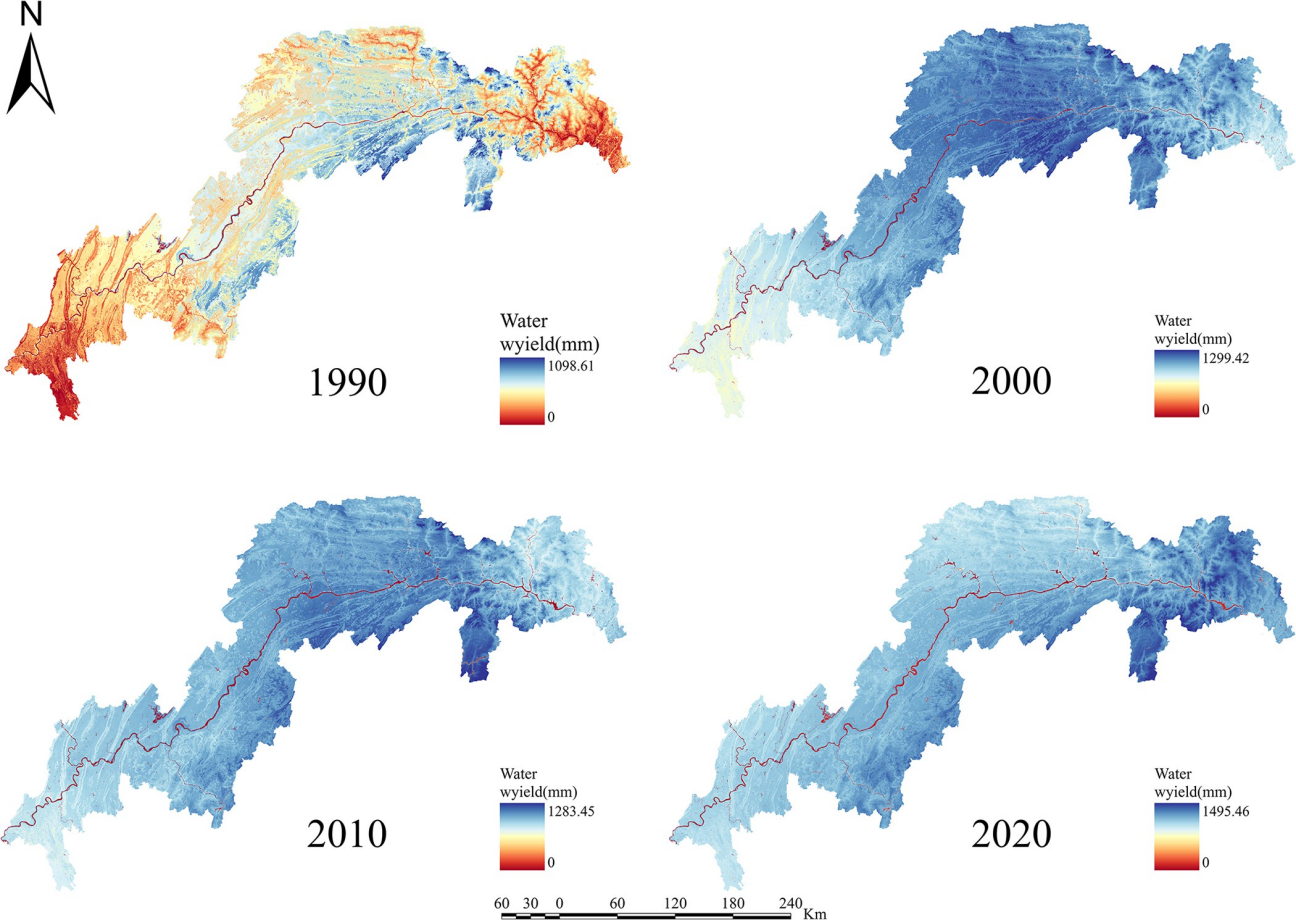
Water yield chart of the TGRA from 1990 to 2020. Calculated by INVEST modeling for water conservation in the Three Gorges Reservoir Area, 1990–2020.

**Fig 4:**
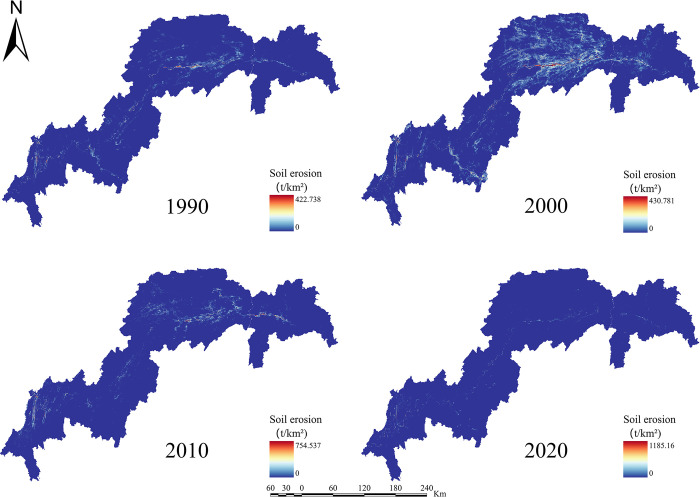
Soil retention map of the TGRA from 1990 to 2020. Calculated by INVEST modeling for Soil Retention in the Three Gorges Reservoir Area, 1990–2020.

**Fig 5:**
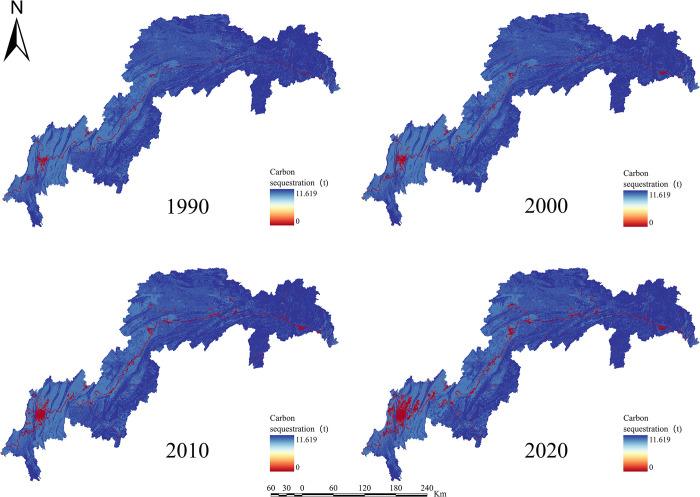
Carbon sequestration chart of the TGRA from 1990 to 2020. Calculated by INVEST modeling for Carbon Sequestration in the Three Gorges Reservoir Area, 1990–2020.

**Fig 6:**
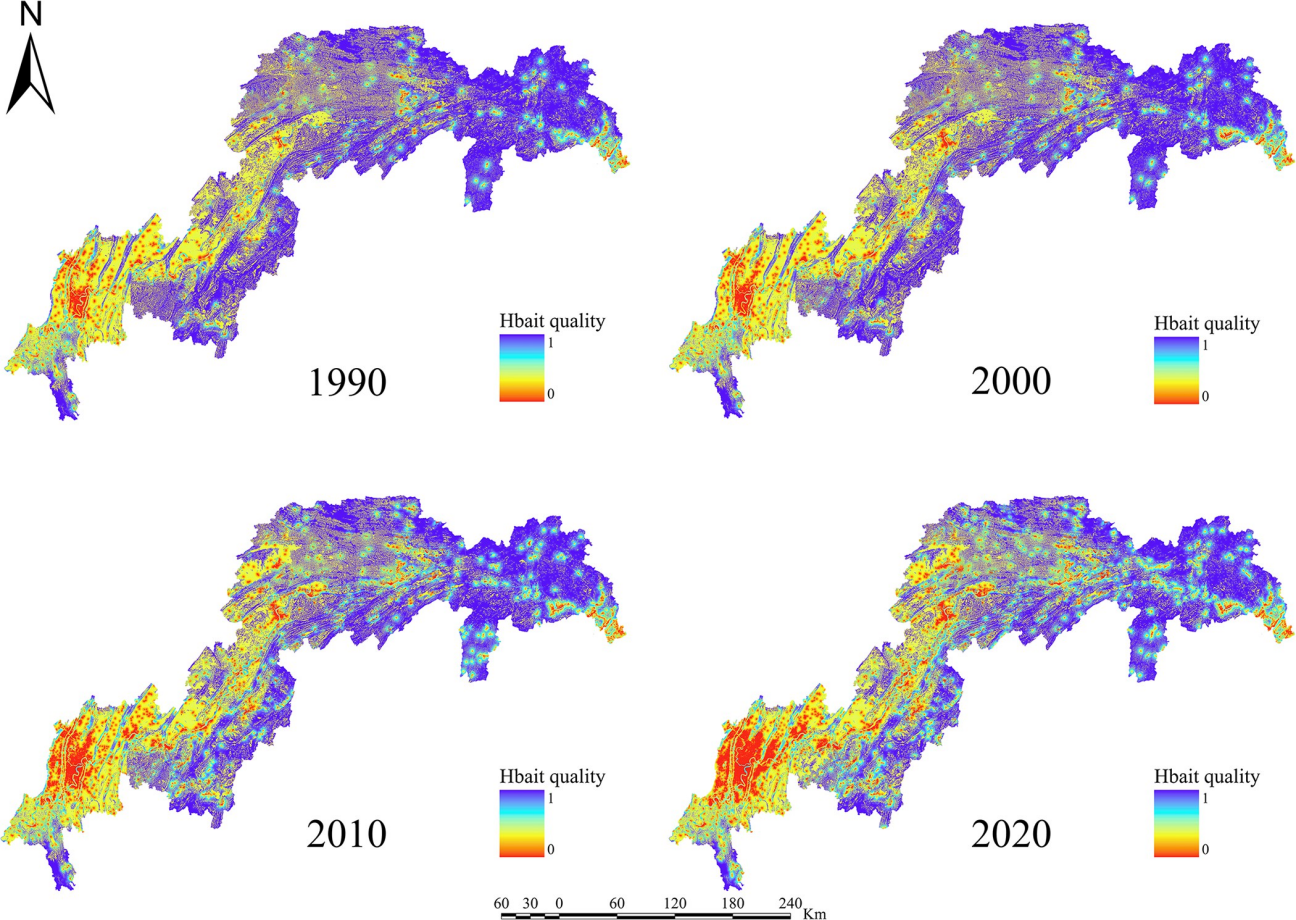
Habitat quality map of the TGRA from 1990 to 2020. Calculated by INVEST modeling for Habitat Quality in the Three Gorges Reservoir Area, 1990–2020.

**Fig 7:**
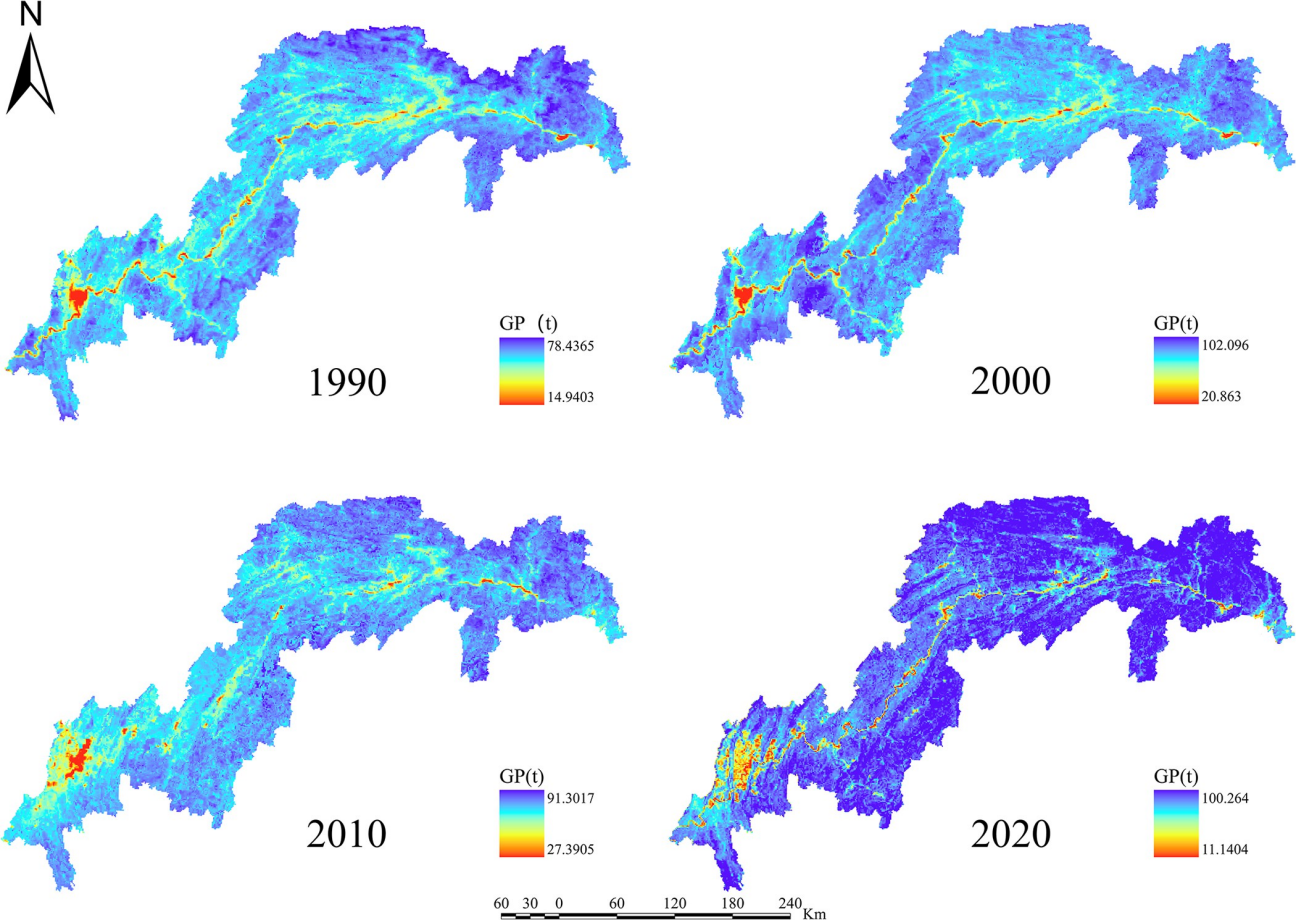
Grain production chart of the TGRA from 1990 to 2020. Calculated by NDVI for Grain Production in the Three Gorges Reservoir Area, 1990–2020.

**Fig 8:**
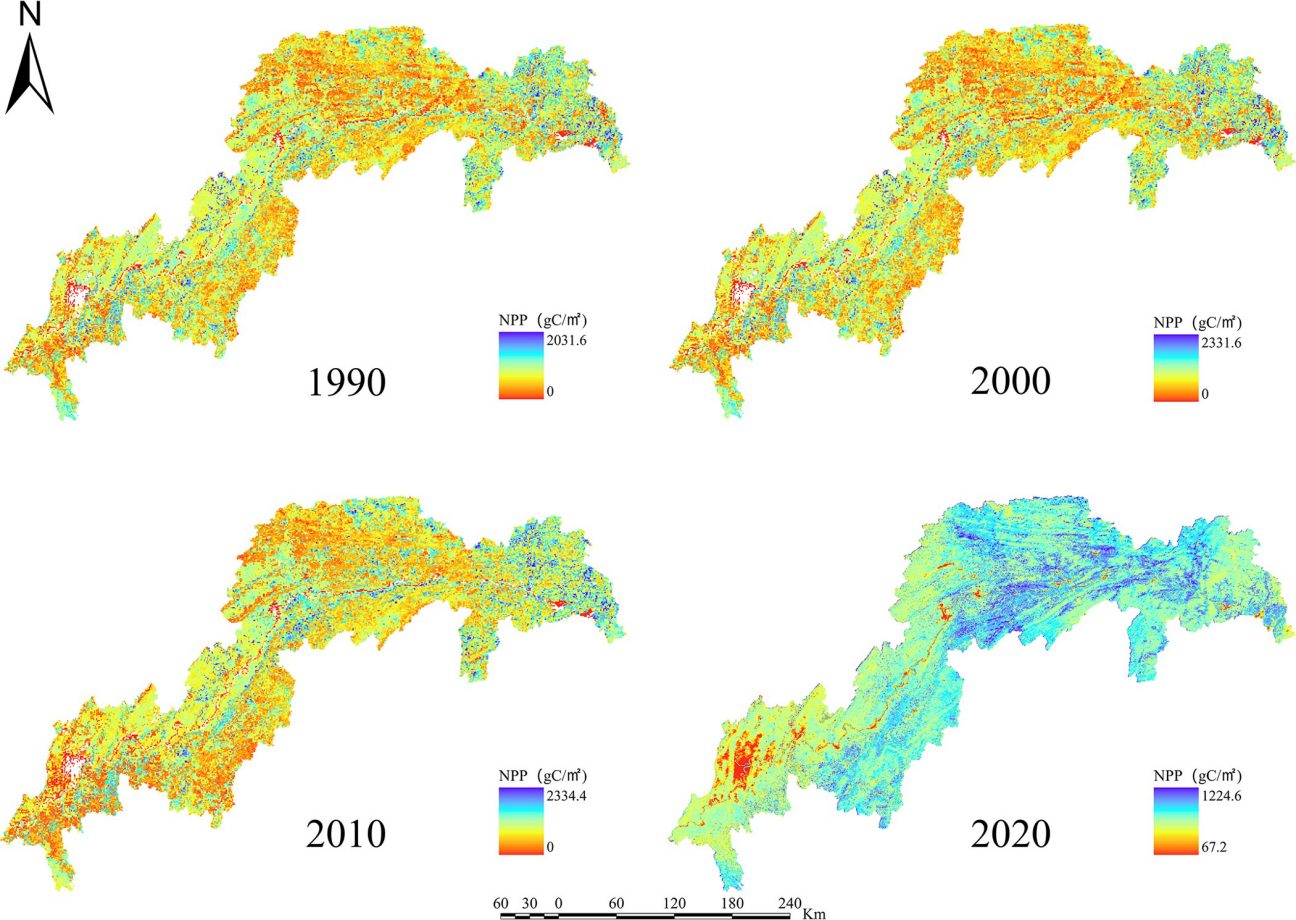
NPP map of the TGRA from 1990 to 2020. Calculated by CASA modeling for NPP in the Three Gorges Reservoir Area, 1990–2020.

For the years 1990, 2000, 2010, and 2020, the ESs efficiency for water yield, soil retention, carbon sequestration, habitat quality, grain production, and net primary production in the TGRA was estimated (Figs 3–8). The time series analysis results demonstrate that throughout the course of 30 years, the TGRA’s water conservation service showed a tendency of early suppression followed by an increase. Over a 20-year period, it witnessed a fall from 1990 to 2000, followed by an increase trend from 2000 to 2020.Over a 30-year period, the quantity of soil erosion increases, followed by a reduction. Over a 20-year period, it showed a rising trend from 1990 to 2000, followed by a decreasing trend from 2000 to 2020. The water yield declined from 6.39×10^8^m^3^ in 1990 to 5.32×10^8^m^3^ in 2000 before increasing to 6.16×10^8^m^3^ in 2020. The quantity of soil erosion grew from 1.35×10^5^t·km^-2^ in 1990 to 1.55×10^5^t·km^-2^ in 2000 and then declined to 1.32××10^5^t·km^-2^ in 2020. Over time, the reservoir area’s carbon sequestration service has steadily increased. For 1990, 2000, 2010, and 2020, the carbon sequestration values are 535.32 g/m^2^, 552.64 g/m^2^, 579.54 g/m^2^, and 616.20 g/m^2^. Changes in habitat quality and grain production, on the other hand, are negligible over a 30-year period. The NPP has been declining over time, with values of 42.98 tC, 41.70 tC, 40.91 tC, and 46.75 tC in 1990, 2000, 2010, and 2020, respectively. In terms of the whole region, the NPP values show a decreased overall spatial average from 1990 to 2010, with the highest NPP value increasing year by year. The overall NPP values rose from 2010 to 2020, but the greatest NPP value fell.

In terms of spatial distribution, the water yield in the TGRA reveals that high-value and low-value areas are concentrated in Badong and Zigui regions in the reservoir’s upper reaches and Wushan, Fengjie, and Yunyang regions in the reservoir’s midsection. From 2000 to 2010, considerable declines in water conservation capacity were recorded in regions such as Kaizhou, Wanzhou, and Yunyang. In 2020, the low-value area for water conservation in Chongqing’s main urban area will continue to expand. Nevertheless, the average value of water conservation services in the study region has grown since 2010, owing to a substantial increase in the geographical distribution of high-value water conservation areas. High-value areas for soil erosion services are found in Shizhu, Wuxi, Badong, and Zigui, whereas low-value areas are found mostly in Shizhu, Wulong, and Fengdu in the reservoir’s centre part. Soil erosion services in the middle and northern regions of the TGRA, such as Jiangjin, Changshou, and Wanzhou, deteriorated significantly in 2010 compared to 2000. The overall geographical distribution of soil erosion in the reservoir region had decreased by 2020 compared to 2010, while the total values had increased. In the TGRA, the geographical distribution pattern of carbon sequestration services is relatively regular. With the Yangtze River as a barrier, the north-western section had lower carbon sequestration values in 2000, while the southeastern and southern sections had greater values. Between 2010 and 2020, the reservoir area’s carbon sequestration capacity expanded dramatically, and the discrepancies on both banks of the Yangtze River decreased. From 2000 to 2020, the spatial distribution pattern of habitat quality services remained mostly consistent, with a modest decline in values in 2020. High-value areas are mostly found in Shizhu, Wulong, and Wuxi and are distinguished by limited development and high habitat quality. Low-value areas are mostly found on the reservoir’s western shore in Jiangjin, Chongqing’s main metropolitan centre, and Changshou. From 1990 to 2000, the geographical distribution pattern of grain production in the TGRA remained substantially similar, with the overall yield distribution maintained steady. However, grain production distribution changed from 2000 to 2010, with per-unit yields being evenly divided on both banks of the Yangtze River, resulting in an overall rise in production. From 2010 to 2020, global grain production grew, while per-unit grain production climbed significantly in several locations. The following are the spatial distribution features of NPP services in the reservoir area: High-production zones are mostly found in the reservoir’s upper reaches, which include sections of Badong, Xingshan, and Zigui, as well as in the reservoir’s middle region, which includes parts of Zhong County, Kaizhou, and Changshou. There was no general change in the distribution of NPP values between 1990 and 2000, although the maximum per-unit area NPP rose. Between 2000 and 2010, the region with low NPP values grew in the reservoir’s middle and lower parts. The total NPP values in the reservoir area from 2010 to 2020.

### 4.3. Analysis of land use simulation results

#### 4.3.1. Analysis of changes in land area for different scenario settings in land use simulation

**Fig 9:**
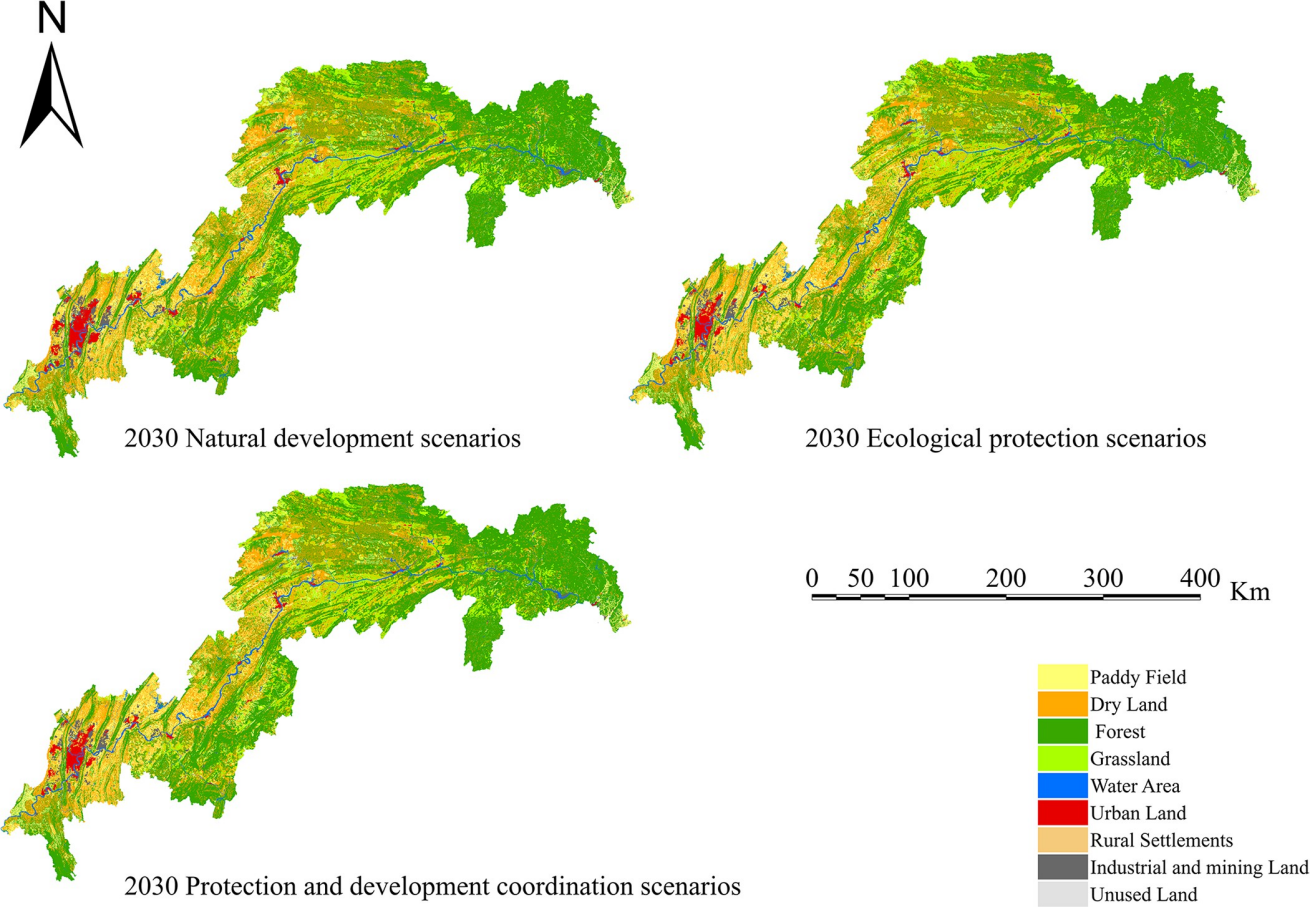
Land use type simulation map for the TGRA in 2030 under different scenario settings. Land use future simulation of the Three Gorges Reservoir area under different scenarios through the FLUS model.

Fig 9 depicts the land use simulation results for the TGRA in 2030 under various scenario scenarios. Under the natural development scenario, forest land rises by 297.64 km^2^; grassland reduces by 180.09 km^2^, and water area increases by 23.84 km^2^ due to a lack of limitations. Overall, ecological land is rising, although grassland is decreasing, with a 2.90% loss rate. Construction land expands by 329.86 km^2^, whereas paddy fields and dryland shrink by 150.69 km^2^ and 319.97 km^2^, respectively. In general, under the natural development scenario, both ecological and economic land areas rise and shrink, with no discernible trend of change.

More farmland with a slope greater than 25° has been designated for afforestation under the ecological protection scenario, resulting in the following simulation results: forest land rises by 570.41 km^2^, grassland increases by 44.32 km^2^, and farmland reduces by 269.37 km^2^. Moreover, building land expands by 89.65 km^2^. According to the simulation data, there is more support for ecological engineering buildings under the ecological protection scenario, with certain restrictions on urban construction. This strategy helps the region to reap greater ecological advantages.

The simulation results show the following changes under the Protection and development coordination scenarios, which are based on the three-line restrictions of ecological protection, permanent basic farming, and urban development borders in the research area: Due to water project limits and the objective of food security, forest land expands by 339.73 km^2^, grassland expands by 44.47 km^2^, while water bodies and paddy fields remain stay unaltered. Construction land expands by 353.15 km^2^. The orderly growth of construction land also provides regional economic advantages to some extent. As a result, this model provides both high-level protection and high-quality development.

#### 4.3.2. Analysis of spatial distribution of different land use types in land use simulation under various scenario settings

Setting constraintrequirements, transition probability matrices, building appropriate regions, and inputting the optimal goal land cover amounts in the GeoSOS-FLUS program resulted in geographical distribution maps for the three scenarios. Except for the natural development scenario, the other two scenarios take into account the actual functioning of food security and hydropower projects. As a result, water bodies and paddy fields stay constant in these two optimum situations. According to the scenario settings, unused land in the economic growth scenario is transformed into built-up areas, forest land in the ecological preservation scenario, and both in the protection and development coordination scenario. As a result, LULC pattern alterations occur mostly in the "Forest-Grassland-Built-up-Dryland" categories in the two optimum scenarios.

Under the natural growth scenario, the increase of economically favourable LULC types is most noticeable, with built-up regions being the most prevalent. The primary regions of growth are in Chongqing’s main metropolitan area and the urban cores of neighbouring districts and counties. Expansion happens on the outside of cities, whereas infill development occurs within cities. Under the ecological protection scenario, there is a large increase of ecologically beneficial LULC types, which are concentrated in the north-eastern section of Kaizhou, adjacent hilly areas within Wuxi, and some locations in Xingshan, Hubei. Forestland and grassland are the two primary types being converted. In the context of urban-rural integration, the pattern of LULC conversion displays a greater emphasis on environmental advantages. Similarly, it occurs in a concentrated and continuous fashion across the reservoir region, mainly in the north-eastern hilly areas of Kaizhou, Wuxi, the northern half of Badong, and Xingshan. Forest land is the most common kind being converted. There is also considerable grassland conversion in Fuling.

In conclusion, the combination of a multi-objective genetic algorithm and the FLUS model allows for the optimization of LULC structure and quantity, as well as spatial distribution simulation, resulting in a more visually comprehensible depiction of the landscape. Notably, in locations significantly impacted by human activity, LULC patterns with considerable economic benefits tend to convert. LULC patterns with significant ecological advantages, on the other hand, are largely modified in places that are concentrated, continuous, and at higher altitudes.

### 4.4. Analysis of ESs efficiency based on future LULC patterns

**Fig 10:**
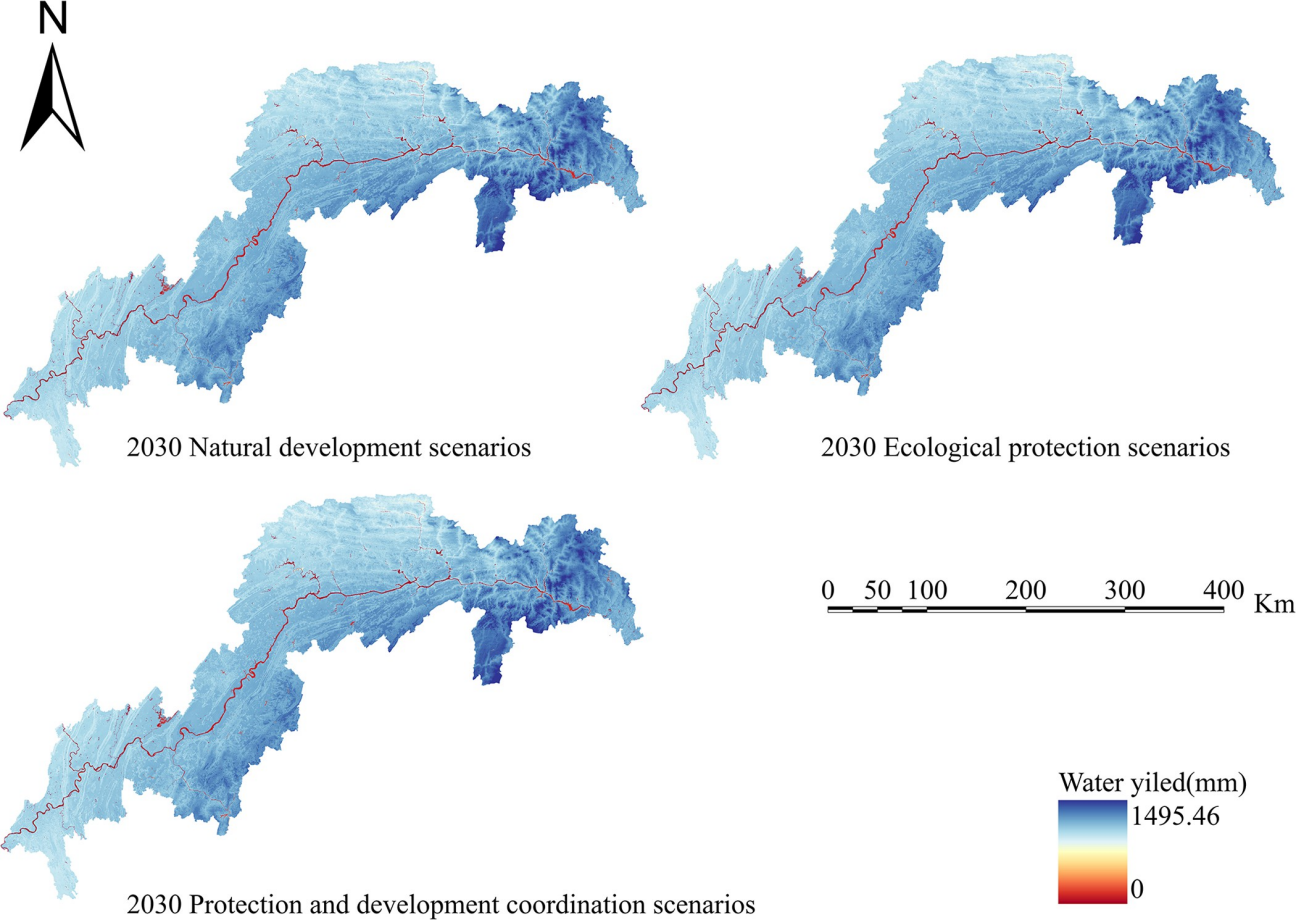
Water yield chart for the TGRA in 2030 under different scenarios. Land use results from FLUS model simulations and INVEST model results for future Water yield.

**Fig 11:**
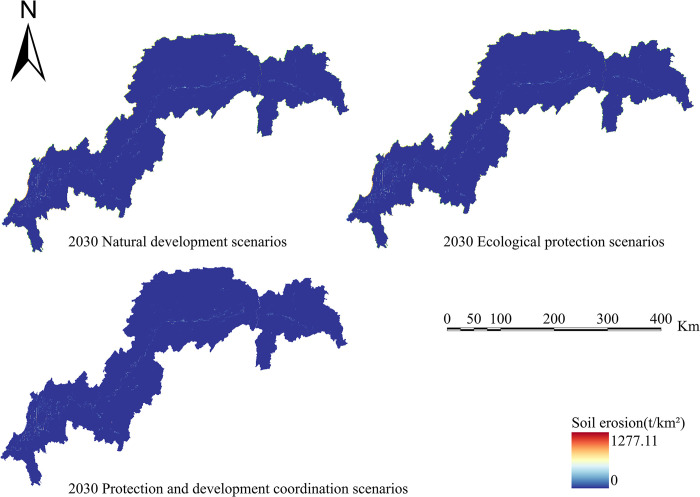
Soil retention chart for the TGRA in 2030 under different scenarios. Land use results from FLUS model simulations and INVEST model results for future Soil retention.

**Fig 12:**
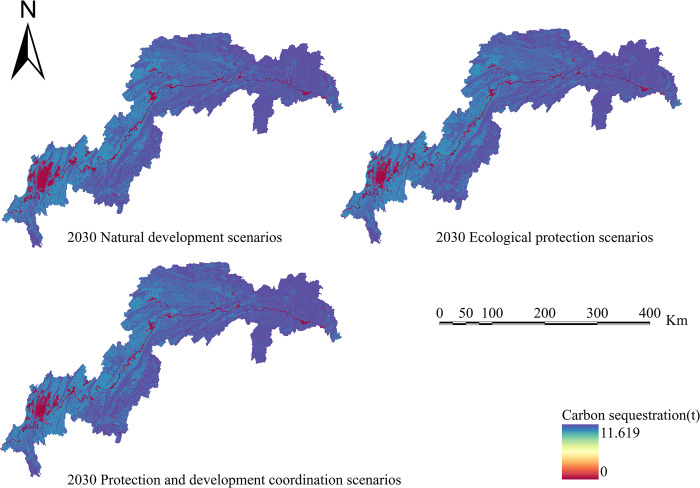
Carbon sequestration chart for the TGRA in 2030 under different scenarios. Land use results from FLUS model simulations and INVEST model results for future Carbon sequestration.

**Fig 13:**
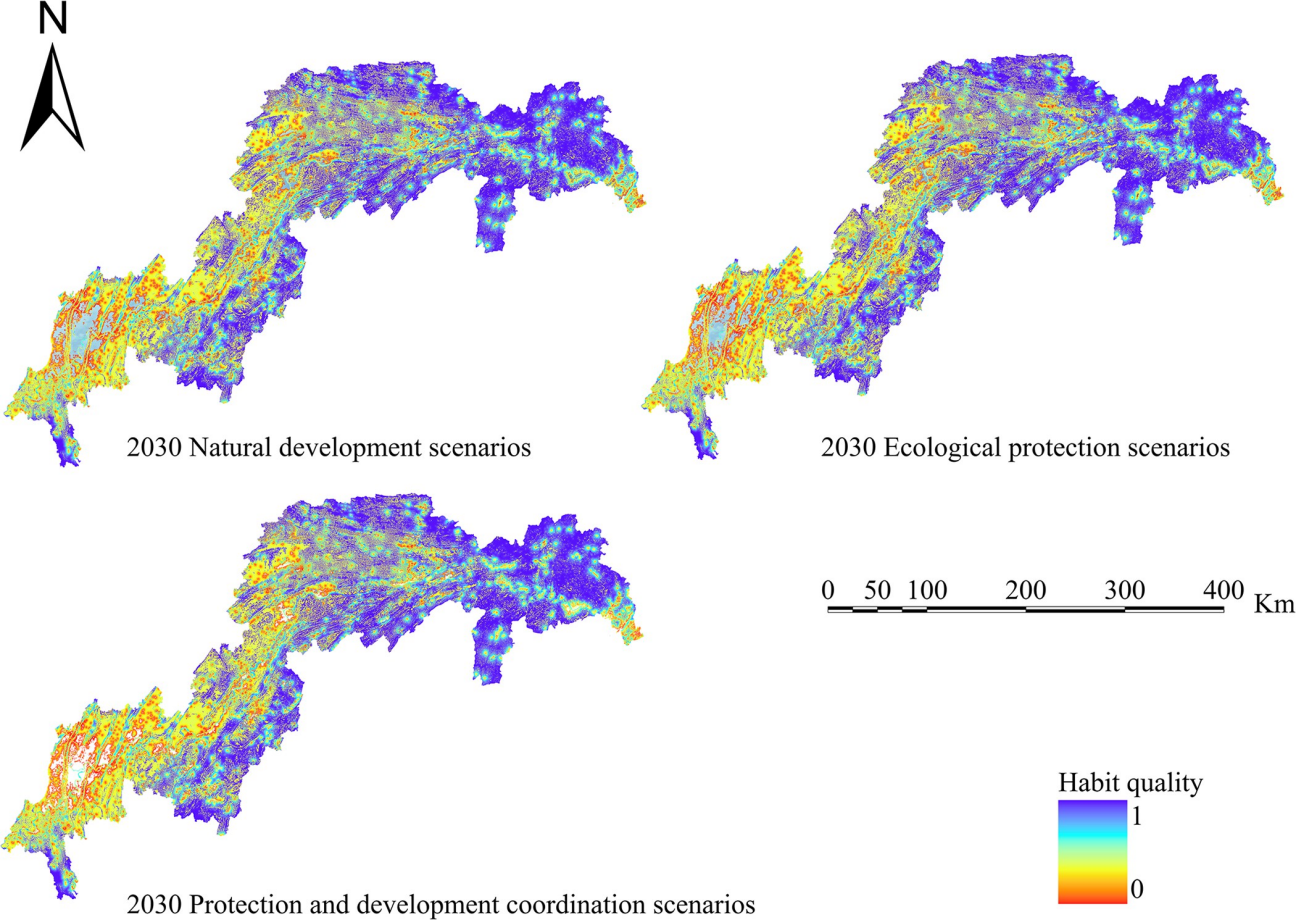
Habitat quality chart for the TGRA in 2030 under different scenarios. Land use results from FLUS model simulations and INVEST model results for future Habitat quality.

Calculate four ecosystem services based on the simulation results of land use in various scenarios: water conservation, habitat quality, carbon sequestration, and soil erosion. Create spatiotemporal distribution maps of the following ecosystem services’ efficiency (Figs 10–13).

The efficiency of water yield in 2030 is shown for three alternative LULC patterns. The low water yield region has reduced in comparison to 2020; however, due to a large rise in the geographical distribution of high water yield, the average water conservation service in the study area is predicted to increase in comparison to the 2020 average value (6.16×10^8^m^3^). The water yield in 2030 is expected to be 6.25×10^8^m^3^, 6.63×10^8^m^3^, and 6.43×10^8^m^3^ under the natural scenario, ecological scenario, and protected development scenario, respectively. Places with poor water conservation values are mostly concentrated in Chongqing’s major metropolitan region, whereas places with high water yield values are mostly concentrated near the reservoir’s head.

The effectiveness of soil erosion services in 2030 is shown for three alternative LULC patterns. For the year 2020, the soil erosion values were 1.32×10^5^ t/km^2^. Soil erosion values in 2030 are expected to be 1.29×10^5^ t/km^2^, 1.25×10^5^ t/km^2^, and 1.26×10^5^ t/km^2^, respectively, under the natural scenario, ecological scenario, and protective development scenario. The regional spread has shrunk in comparison to 2020, while the total numerical values have risen. In 2030, sections including Jiangjin, Changshou, and Wanzhou in the reservoir’s middle and northern reaches see soil conservation service deterioration compared to 2020.

The following are the carbon storage figures for 2030 under three distinct LULC patterns: Under the natural development scenario, prospective carbon storage in the TGRA is lowered from 615.92 g·m^-2^ in 2020 to 595.62 g·m^-2^ in 2025. Future carbon storage values in the ecological conservation scenario and the integrated protection and development scenario are higher than in 2020, rising from 615.92 g·m^-2^ to 635.24 g·m^-2^ and 626.32 g·m^-2^, respectively.

The effectiveness of habitat quality services in 2030 is shown for three alternative LULC patterns. In terms of spatial distribution, the change trend is rather minor in comparison to 2020. High-value areas are mostly found in Shizhu, Wulong, and Wuxi, whereas low-value areas are mostly found on the west side of the reservoir area in Jiangjin t, Chongqing’s major metropolitan centre, and Changshou.

### 4.5. Analysis of the impact of land use patterns on the ESs efficiency

#### 4.5.1. Impact of 1990–2020 LULC pattern changes on ESs efficiency

The Three Gorges Water Conservancy Hub Project began in December 1994, involving the relocation of urban and rural residents in flooded areas along the Yangtze River, as well as the occupation of ecological land such as cultivated land, forests, and grasslands for infrastructure development. This resulted in an increase in construction land area from 4.08×10^8^ m^2^ in 1990 to 23.86×10^8^ m^2^ by 2020. The middle and western areas of Chongqing’s main urban areas, including Jiangbei, Yubei, Shapingba, and Jiulongpo, are the most impacted. On the one hand, growing urbanization encroaches on ecological land, reducing the effectiveness of ESs. Construction and growth of metropolitan areas, on the other hand, entail the consumption of food, carbon emissions, energy, water resources, and so on, all of which have a substantial negative influence on the regional efficiency of ESs. The spatial characteristics of the reservoir area show a decrease in cultivated land area of 14.1×10^8^ m^2^, a decrease in grassland area of 19.08×10^8^ m^2^, a decrease in unused land area of 106.11 hectares, an increase in forest land area of 8.72×10^8^ m^2^, and an increase in water area of 4.5×10^8^ m^2^ over the course of 30 years. The rise in forest land area may be ascribed to the TGRA’s conversion of cultivated land to forest since 2002, as well as the efficient implementation of regional environmental preservation and restoration initiatives. The growth of the water area is the outcome of the Three Gorges Reservoir’s phased impoundment, which results in a progressive overall rise in water surface area.

The TGRA’s alterations in LULC patterns have a substantial influence on ESs efficiency. The reservoir region’s grain production capability has decreased from 78t/m^2^ to 62t/m^2^ over 30 years due to a decline in cultivated land area. The increase in forest land area has successfully increased the ecosystem’s potential for carbon storage and sequestration, with carbon storage in the reservoir region increasing from 535.32g/m^2^ to 616.20g/m^2^. NPP has increased from 42.98tC to 46.96tC, helping to promote ESs.

The water yield services in the TGRA have a declining and then increasing pattern. This is due to the construction of the Three Gorges Dam and the relocation of population in the reservoir region, which resulted in the conversion of a major section of forests and grasslands in various districts and counties into agricultural and construction land. As a result, water conservation services in the research region have significantly declined. Nonetheless, with the impoundment of the Three Gorges Dam, the water surface area has increased. Furthermore, since 2012, the reservoir region has actively undertaken initiatives, including the conversion of farmland to forests and grasslands, the establishment of green belts in the reservoir area, and the restoration of farmland to forests and grasslands. As a result, vegetation covering has improved, and total water conservation service capacity has steadily increased.

The changes in soil erosion services follow the same pattern as those in water yield services, with an initial reduction followed by an increase. This is strikingly similar to Tian Yu’s and others’ study findings. In 2010, the regions with poor soil erosion services were predominantly concentrated in the central reservoir area, which is connected to the region’s rapid expansion of urban building and business. Concurrently, this tendency is linked to precipitation levels. Reduced precipitation in degraded regions in 2010 resulted in a decline in the values of soil erosion services to some extent, driven by the Rainfall Erosivity Factor (R) in the Revised Universal Soil Loss Equation (RUSLE). Although certain places still had deteriorated soil retention services in 2020, generally, improvements in vegetation covering contributed to a rise in the value of soil erosion services.

#### 4.5.2. Analysis of the impact of different scenarios of land use simulation in 2030 on Ess

Following are some of the tendencies that are revealed by the simulation results of three distinct LULC scenarios: In the natural growth scenario, the total area of ecologically useful land, which includes grassland and farms that contribute favorably to ecological sustainability, has declined in comparison to the year 2020. This is despite the fact that the forest area has reached a higher level. Meanwhile, the area of land that is used for development has grown, with the majority of this expansion occurring in the primary urban centre of Chongqing, which is located near the downstream end of the reservoir.

Compared to the year 2020, the ecological system has shown an increase in its capacity to store carbon and manage soil erosion. This is supported by the ecological protection scenario. When compared to the natural development scenario, this scenario produces a greater number of positive effects on the environment. As a result of the conversion of some farmland into environmentally useful land, such as woods and grasslands, the ecological protection scenario leads to a decrease in food production. This is in contrast to the integrated conservation and development scenario, which results in an increase in food production.

**Fig 14:**
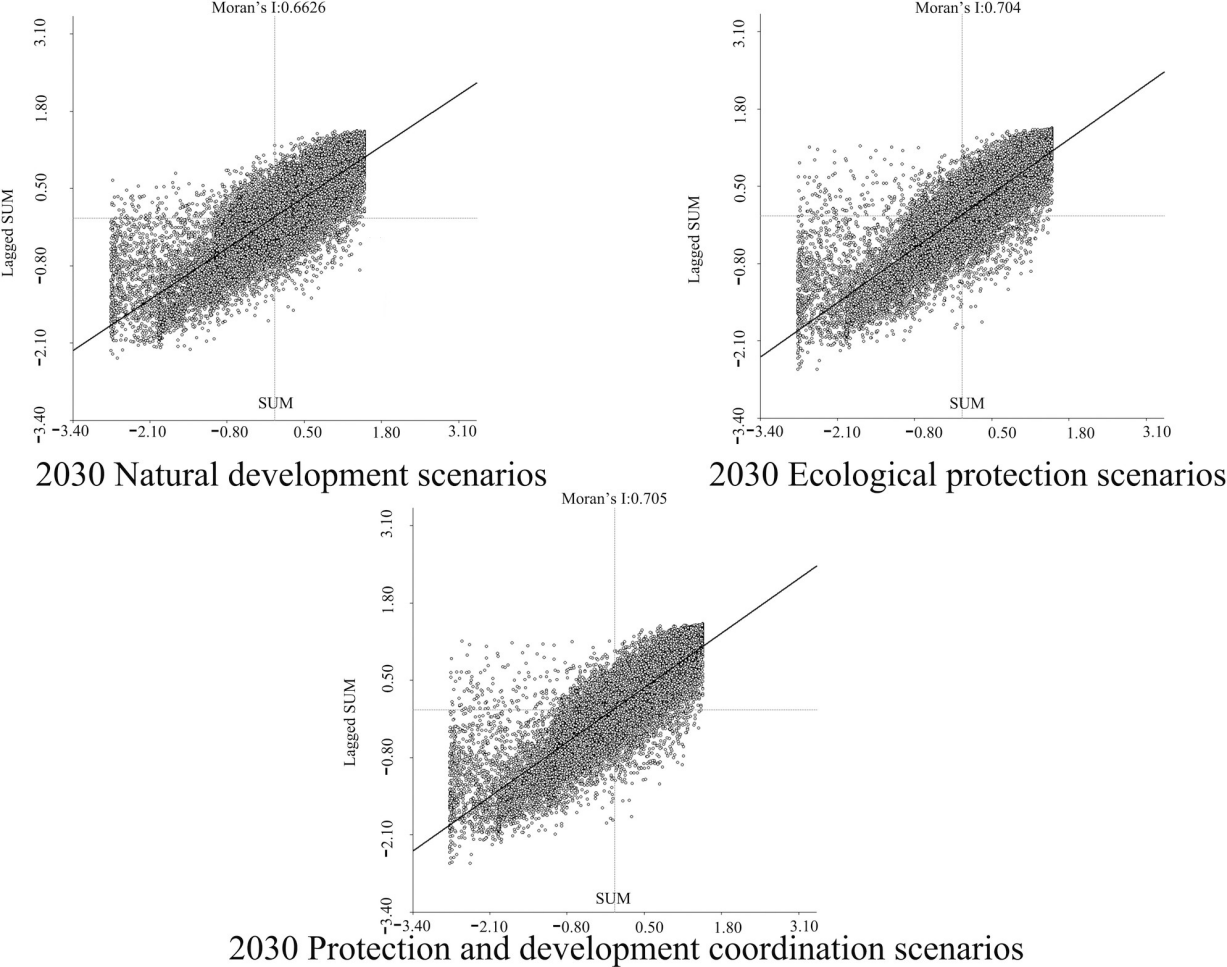
Spatial autocorrelation Moran scatter plot of carbon sequestration in the TGRA in 2030 under different scenarios. Representation of the correlation between carbon sequestration under different scenarios by means of a moran scatter plot.

**Fig 15:**
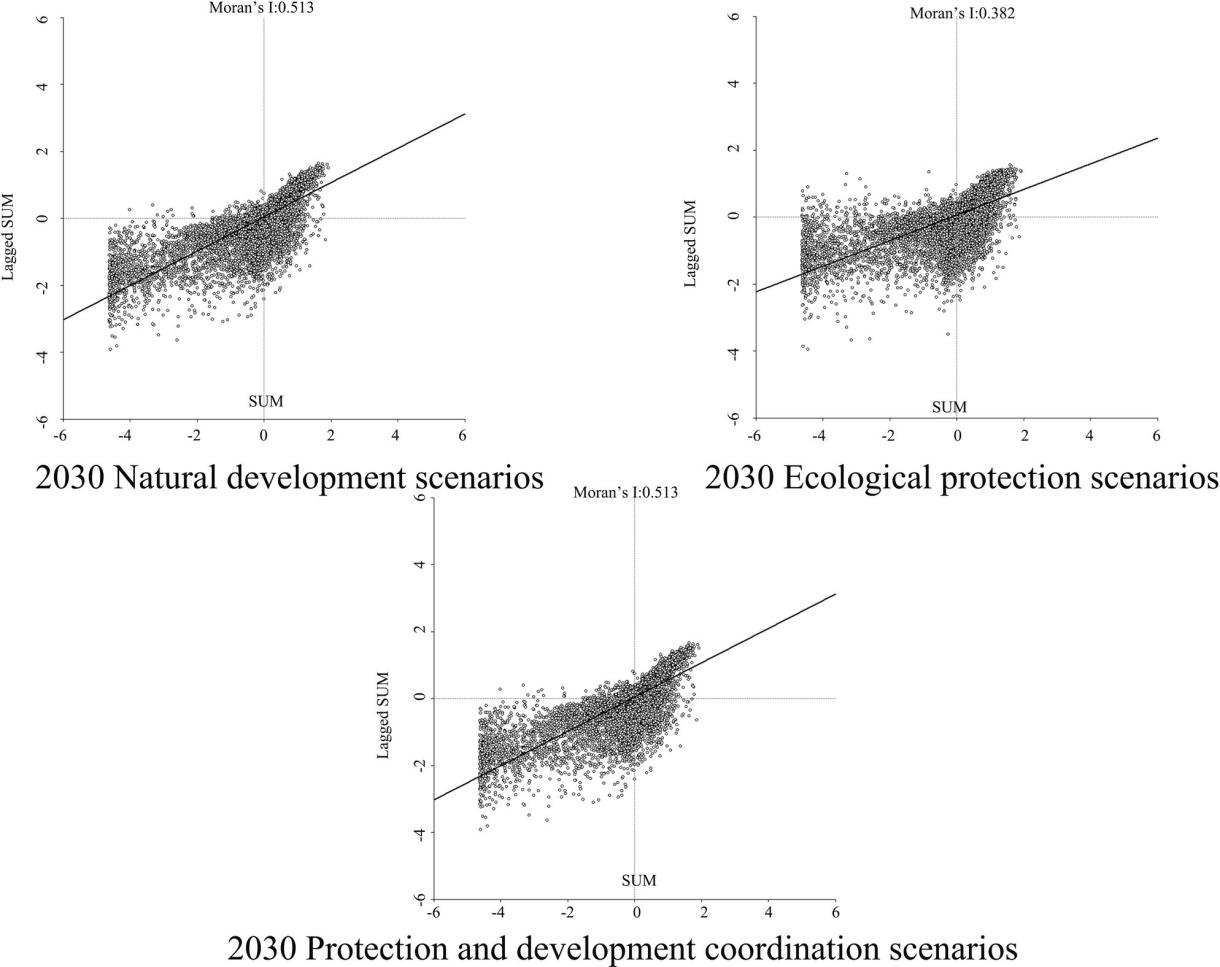
Spatial autocorrelation Moran scatter plot of water yield in the TGRA in 2030 under different scenarios. Representation of the correlation between Water yield under different scenarios by means of a moran scatter plot.

**Fig 16:**
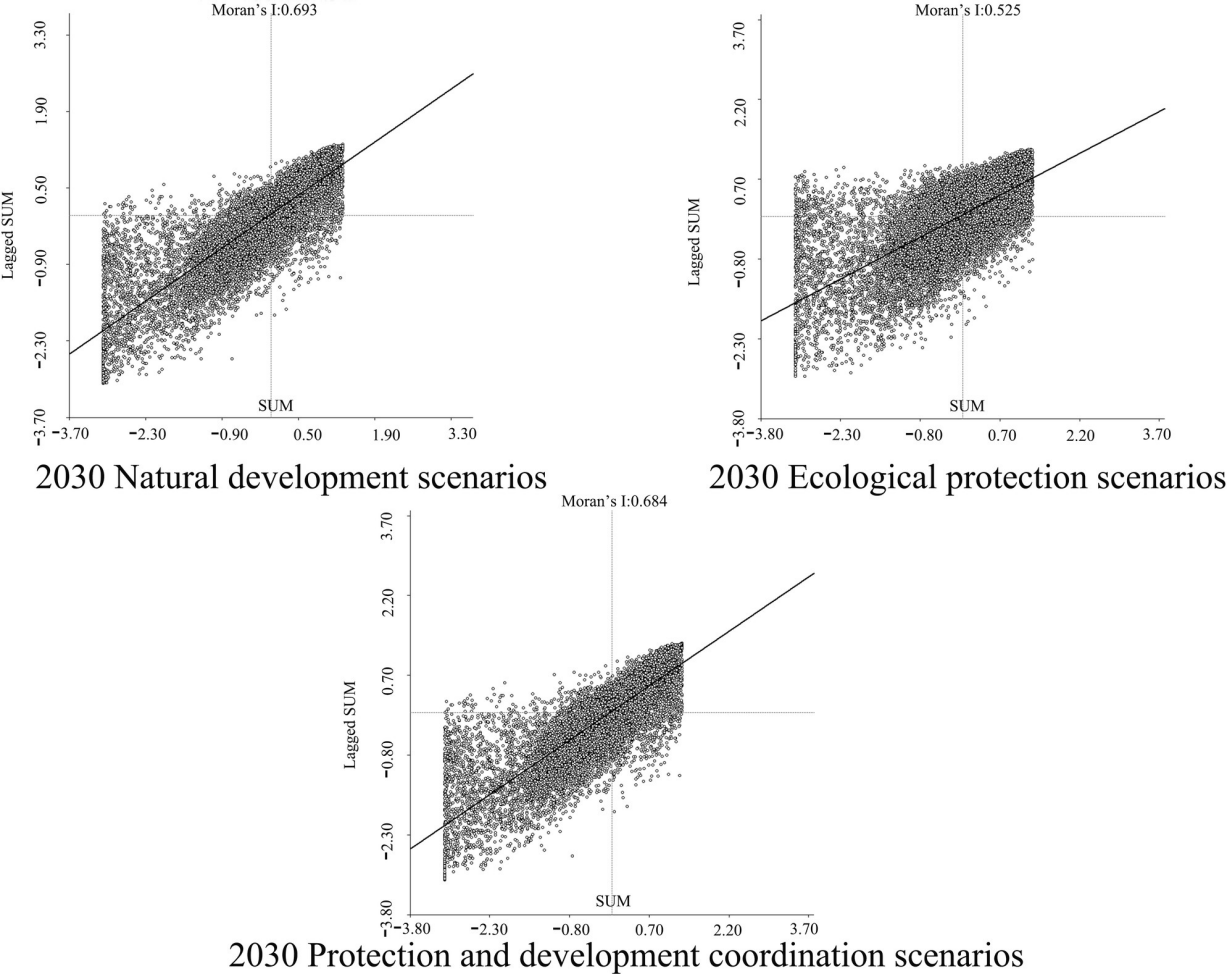
Spatial autocorrelation Moran scatter plot of Habit quality in the TGRA in 2030 under different scenarios. Representation of the correlation between Habit quality under different scenarios by means of a moran scatter plot.

**Fig 17:**
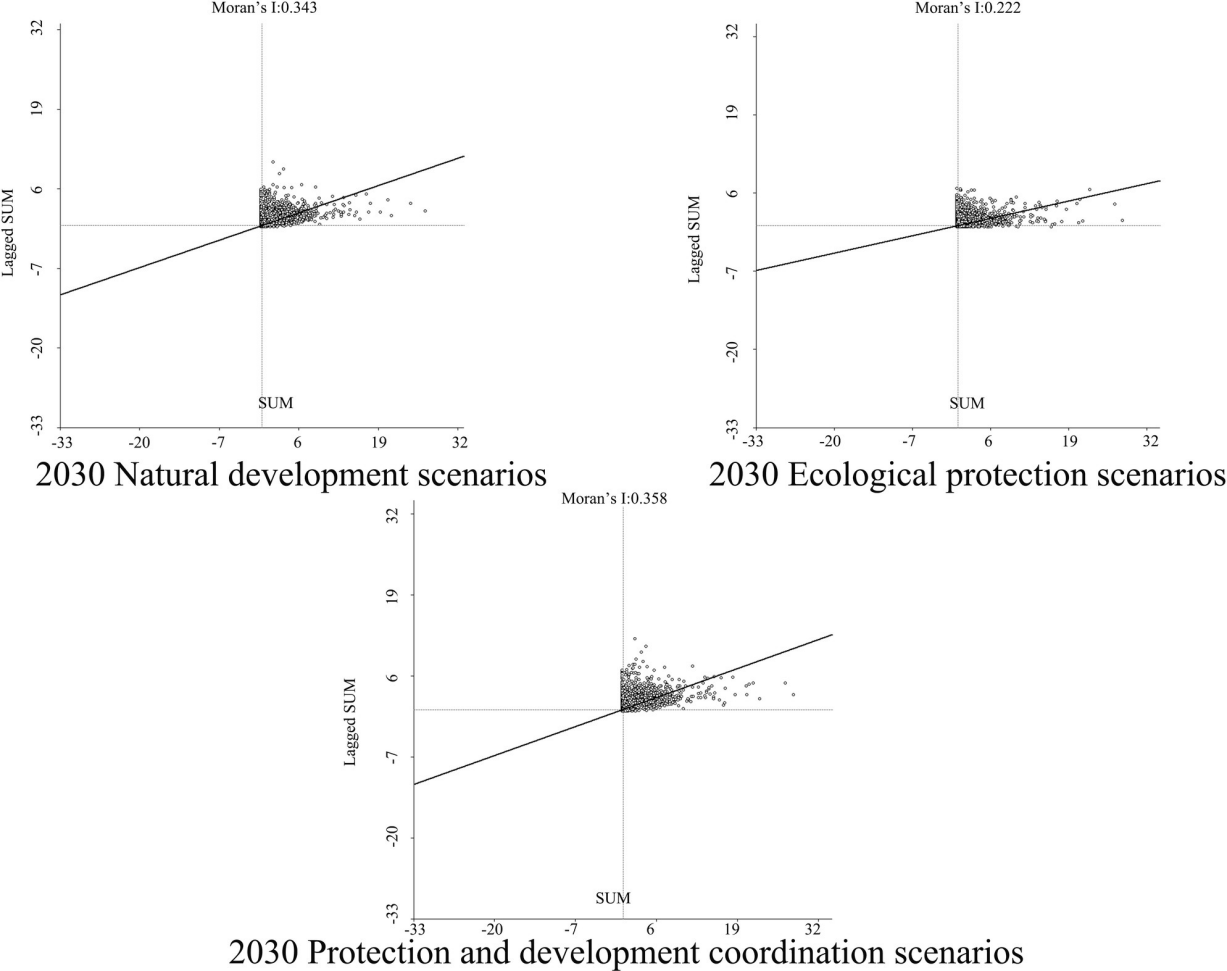
Spatial autocorrelation Moran scatter plot of soil retention in the TGRA in 2030 under different scenarios. Representation of the correlation between Soil retention under different scenarios by means of a moran scatter plot.

**Fig 18:**
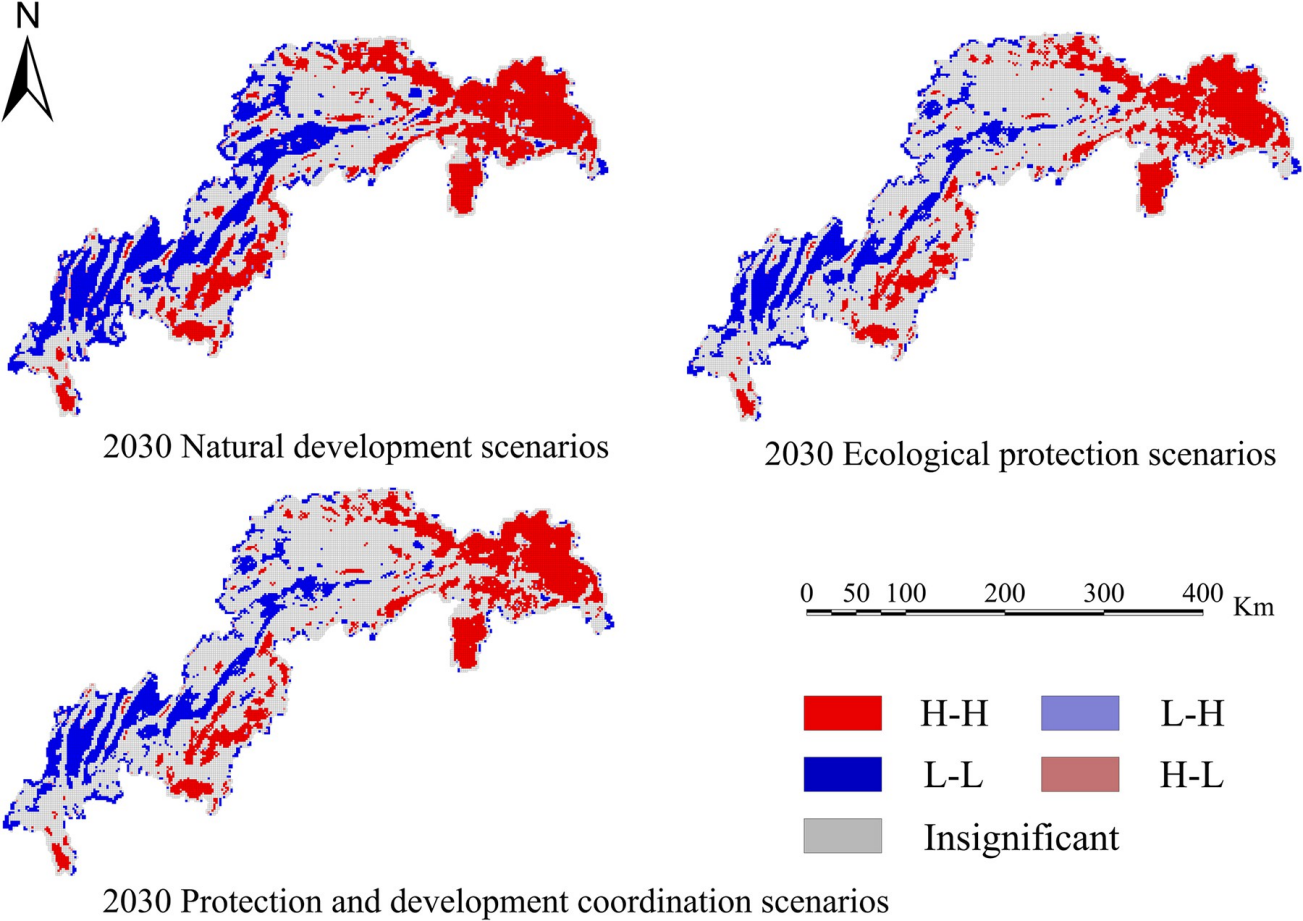
LISA map of carbon sequestration in the TGRA in 2030 under different scenarios. Demonstrating the spatial correlation of Carbon sequestration measured under different future scenarios through LISA clustering.

**Fig 19:**
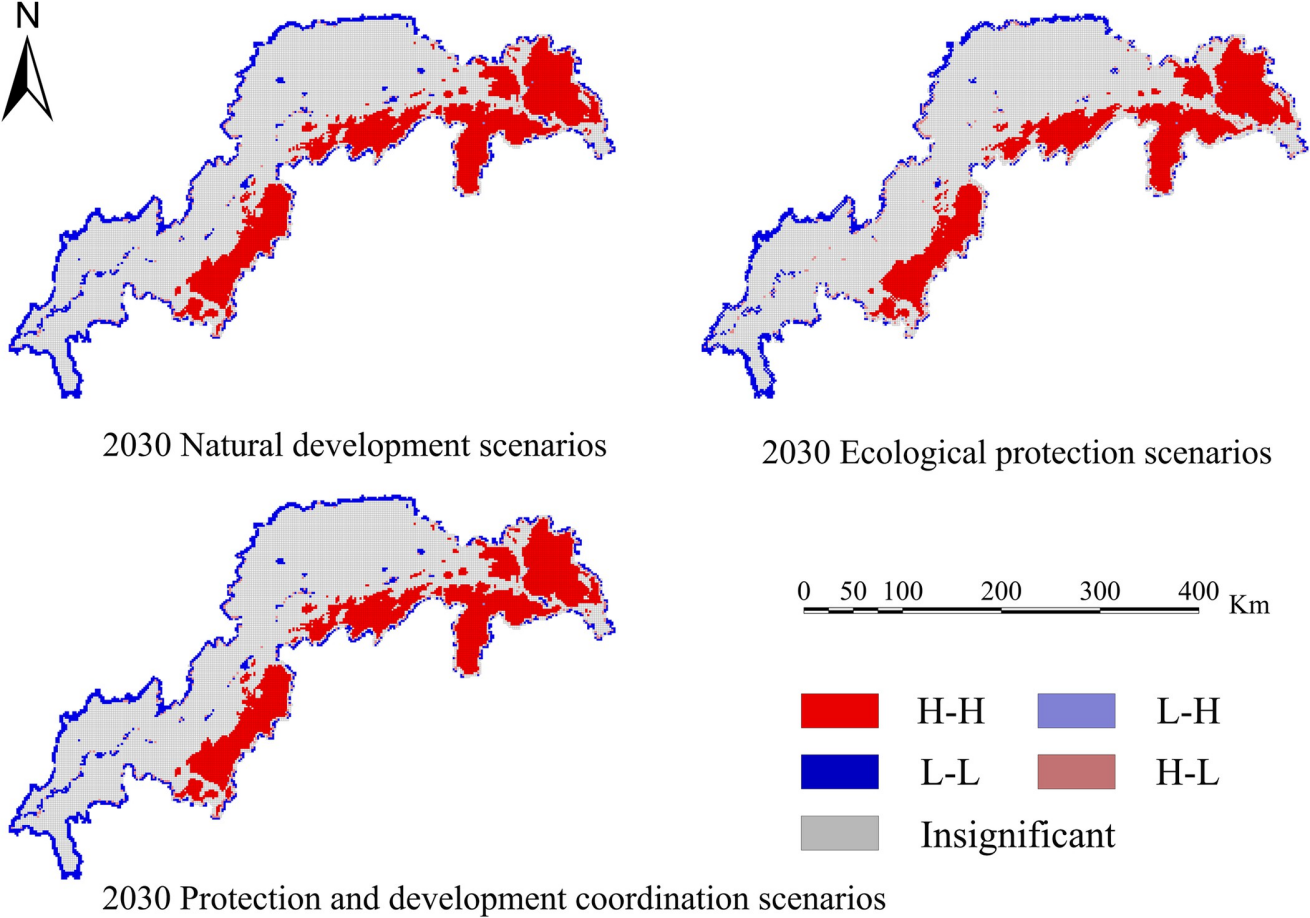
LISA map of water yield in the TGRA in 2030 under different scenarios. Demonstrating the spatial correlation of Water yield measured under different future scenarios through LISA clustering.

**Fig 20:**
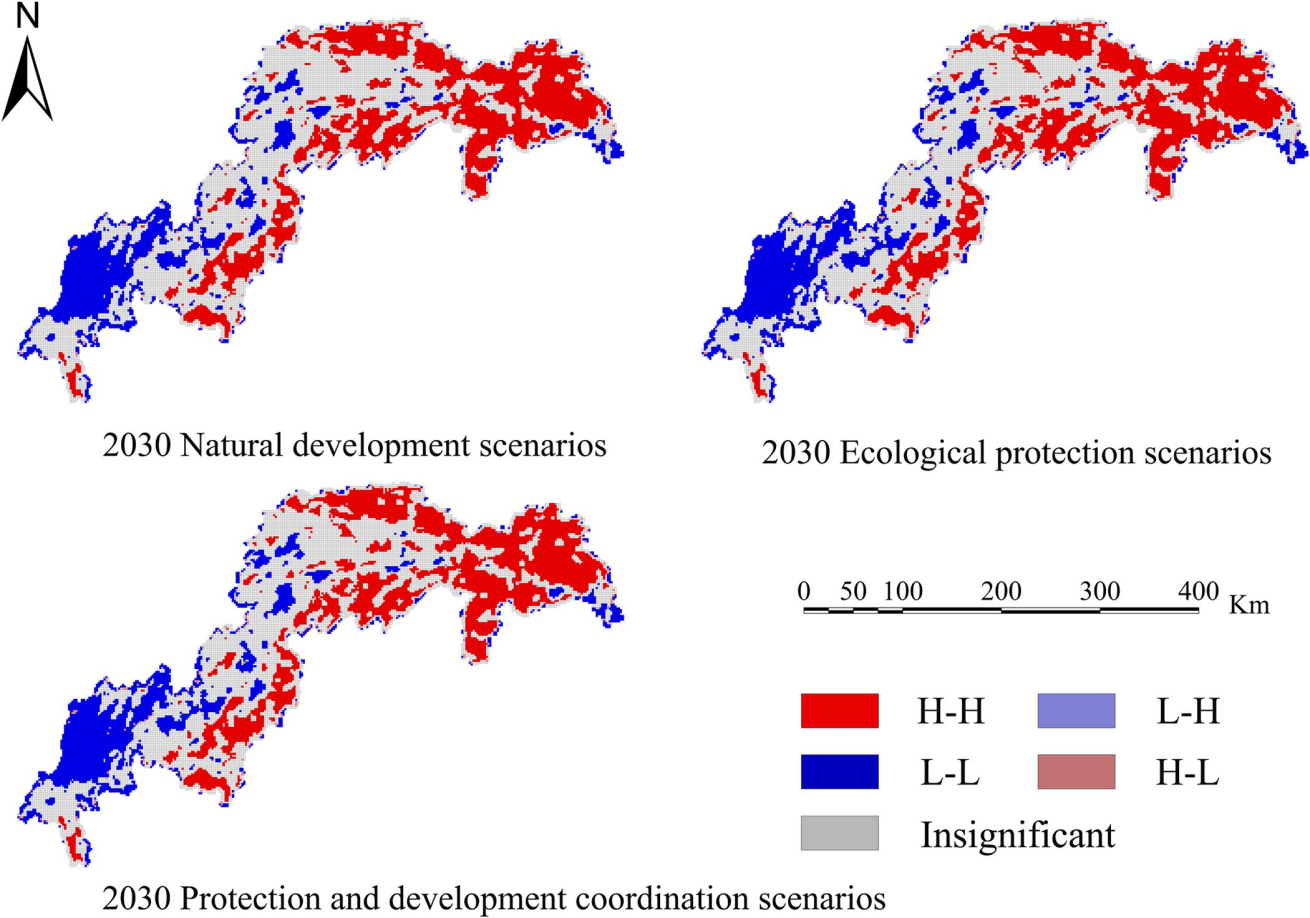
LISA map of Habit quality in the TGRA in 2030 under different scenarios. Demonstrating the spatial correlation of c Habit quality measured under different future scenarios through LISA clustering.

**Fig 21:**
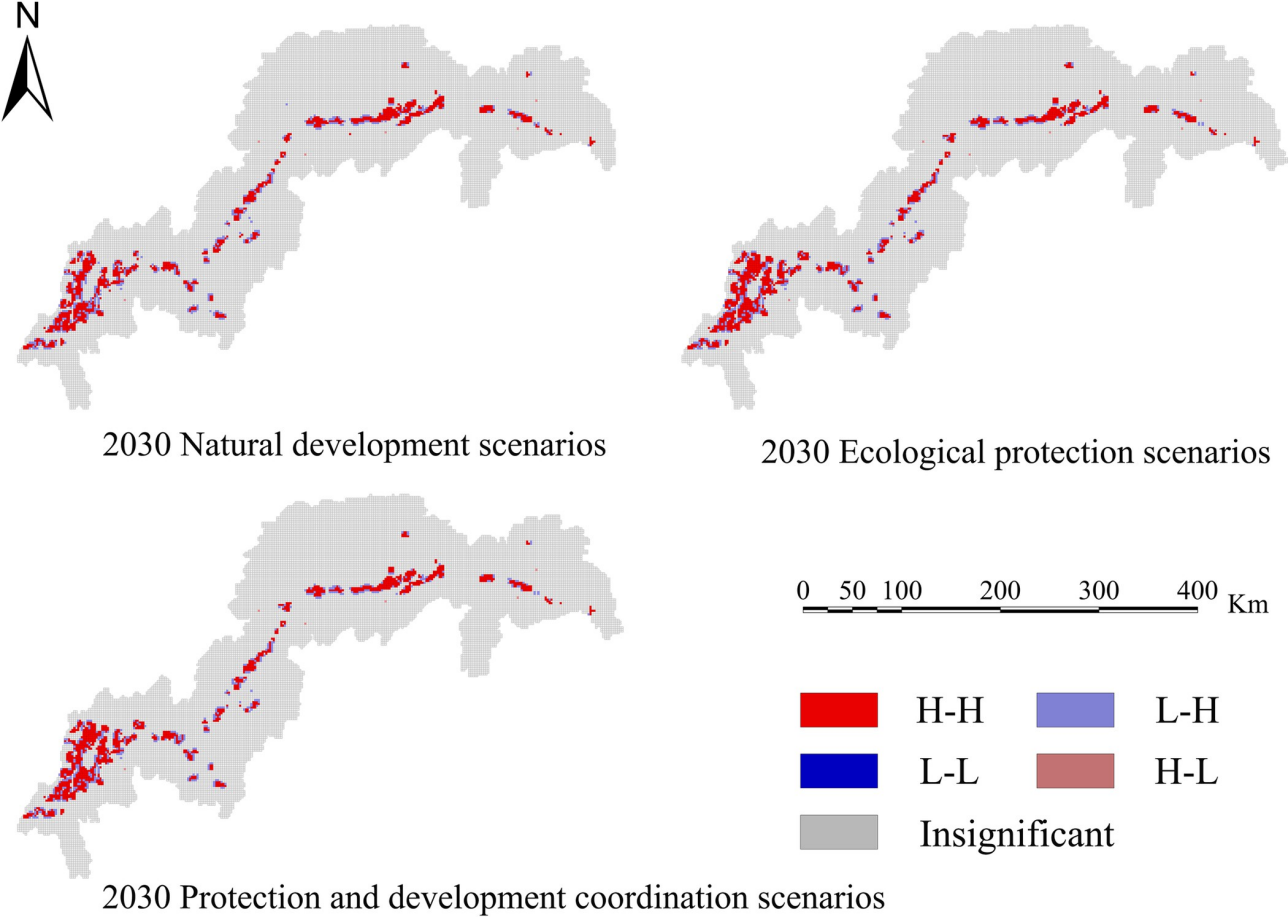
LISA map of soil retention in the TGRA in 2030 under different scenarios. Demonstrating the spatial correlation of Soil retention measured under different future scenarios through LISA clustering.

In order to determine the geographical and temporal disparities of four ecosystem services in the Three Scenarios by the year 2030, a global spatial autocorrelation study was carried out using the Geoda program. This analysis was performed on the administrative divisions of the TGRA. Figs 14–17 display the Moran scatterplots for the four ecological services that are being discussed. The figures demonstrate that there is a geographically positive correlation between the four ecosystem services whenever any of the three scenarios are included. Local spatial autocorrelation analysis was further employed to generate LISA cluster maps for the four ecosystem services (Figs 18–21). Within the TGRA, the geographic correlation features of carbon storage, habitat quality, water yield, and soil erosion were analysed and identified. These qualities were shown to be spatially related. High-value areas of water conservation, soil retention, carbon storage, and habitat quality in the ESs efficiency overlap to some extent, so do the low-value areas.

Within the reservoir region, the first and middle parts are where the majority of the high-value areas of ESs are located. This pattern is due to the fact that certain regions have a greater covering of grasslands and woods than other regions. Afforestation and the conversion of farmland into forest are two examples of ecological protection methods that have been implemented in these regions. These strategies have effectively improved the efficiency of carbon sequestration, water yield, and habitat quality in the local ecosystem. Despite the fact that the TGRA is confronted with obstacles such as frequent geological catastrophes, sensitivity to water conditions, and severe soil erosion, the demarcation of regional ecological protection redlines has effectively controlled the encroachment of ecological land. This has helped to ensure that the TGRA continues to preserve its biodiversity, as well as its water conservation and soil retention efforts.

## 5. Discussion

### 5.1. Promotion of sustainable land use planning in the TGRA for the enhancement of ESs capacity

Changes in LULC patterns are one of the most important variables determining the effectiveness of ecosystem services. Changes in LULC patterns have an influence on the regional ecosystem structure, processes, and functions, lowering the efficiency of regional ecosystem services. The TGRA is a key biological barrier in the Yangtze River Basin, as well as a vital reserve for national freshwater resources. Improving the effectiveness of regional ecosystem services in the TGRA is critical. The objective is to create a reservoir living community out of mountains, water, woods, fields, lakes, and grasses while conserving the authenticity and integrity of the TGRA’s biological system. Coordination of the scientific definition and administration of the three redlines (ecological protection redline, permanent basic farmland protection redline, and urban development boundary) is also critical for the TGRA’s long-term growth.

The TGRA’s sustainable land use planning aims to improve ESs efficiency, optimize the distribution of different land use types, and achieve maximum ESs efficiency, the strongest resource and environmental carrying capacity, and optimal land benefits through the assessment of regional natural resources’ carrying capacity (land, water, flora and fauna, minerals, etc.). As a result, the control of the red line for the protection of permanent basic farmland in the reservoir area, under scenarios of ecological conservation and coordinated development, prevents high-quality arable land and agricultural space from being encroached upon by other socio-economic activities, as demonstrated in this study through quantitative analysis and prediction of future LULC pattern changes. This successfully secures the TGRA’s food production’s long-term expansion. The TGRA is vulnerable to geological disasters, sensitive to water conditions, and a critical region for soil erosion prevention and control. The Three Gorges region’s scientific demarcation and stringent supervision have severely limited encroachment on ecological and agricultural land. This has aided in water conservation, soil retention, and biodiversity preservation in the TGRA, assuring the security of regional food production. Human-environment conflict is important in the TGRA, with urban agglomeration primarily centred along the banks of the Yangtze River in the Yangtze River Basin. The unrelenting pursuit of urban development size at the price of encroaching on agricultural and biological land poses a major danger to the reservoir area’s ESs efficiency in the "high-speed, low-quality" urbanization process. Controlling the urban development boundary effectively prevents disorderly urban expansion, reduces excessive urban development on ecological land, and improves the reservoir area’s ecosystem services, such as water conservation, food production, and habitat quality.

### 5.2. Coordinated strategies for the protection and development of the TGRA

Guided by national policies such as the Yangtze River conservation and the high-quality development of the Yangtze River Economic Belt, the combined efforts of high-level preservation and high-quality development in the TGRA are key components of its sustainable growth in the future. The high-level protection that is provided by the TGRA is systematically directed towards the whole Yangtze River basin. The objective of this protection is to maintain the overall integrity, stability, and efficiency of the ecosystem that is found in the reservoir sector. First and foremost, it is necessary to conduct a scientific evaluation and draw borders for ecological buffer zones, urban building areas, industrial production regions, and other places. Additionally, control mechanisms should be strengthened in order to better safeguard and increase the ecological efficiency of ecological land. The second step is to strengthen the implementation of essential technological research and development for vegetation protection and restoration projects such as the Long-Term Forestation Program, Tianbao Project, and the Conversion of Cropland to Forest Project. In the TGRA, the restoration of the structure and functions of the ecosystem is successfully promoted by the rise in the amount of forestland, grassland, and other forms of land, as well as the balanced spatial distribution of their respective types of land. Also, the efforts that are being made to ensure the safety of the water environment and to restore the ecological system in the TGRA should be intensified. Because there are so many tributaries in the reservoir region, it is important to think of each tributary basin as a separate entity. This will allow for the further consolidation of efforts in river management (the River Chief System) and complete treatment techniques that are suited to one particular river. The geographical arrangement of production, living, and biological areas along the tributaries in the reservoir area should be taken into consideration. Additionally, the governance needs of various regions should be addressed, and an important and representative selection of tributaries should be made for systematic management. In order to encourage the conservation of water resources, improve the quality of the water environment, and increase the functioning of aquatic ecosystems, it is necessary to implement comprehensive measures such as the conservation of soil and water, the protection and restoration of aquatic ecosystems, the connection of rivers, and the development of integrated rural environmental conditions.

It is critical to clarify the fundamental logic of how high-level protection drives high-quality development and how high-quality development leads to high-level protection. This will help to reinforce the foundation for collaborative conservation efforts and the goal of creating a green development demonstration zone where humans and the environment can dwell in harmony. It is critical to adhere to an ecological priority and green development strategy in the reservoir region, embracing the road of industrial ecologicalisation and ecological industrialisation. On the one hand, maintain comprehensive governance, systematic governance, and source control rules based on exact, scientific, and legally sound pollution control principles. Create a strong biological barrier in the Yangtze River basin to help achieve the goals of "peak carbon" and "carbon neutrality," better supporting the region’s high-quality development. The foregoing approaches increase the stability of the reservoir embankment slopes as well as the structure and functions of the terrestrial ecosystem surrounding the reservoir. The goal is to create a healthy and stable ecology around the reservoir, as well as to improve the ecological carrying capacity of the surrounding area. Moreover, it is critical to accelerate the advancement and enhancement of digital infrastructure building to lay a strong foundation for the successful modernization of the TGRA’s ecosystem governance system and governance capability.

### 5.3. Innovation and limitations

Previous TGRA research on LULC and ESs has mostly been focused on present land use patterns, calculating the service capacity of 2–4 types of ecosystems, and so on. However, study that focuses on forecasting future changes in LULC patterns, projecting trends in the efficiency of six ESs, and investigating ESs efficiency enhancement are scarce. Building on standard ESs evaluations, this study integrated two more categories of ecosystem services, namely grain production and NPP. The study adopted a hybrid approach of FLUS model and InVEST, RUSLE, CASA, etc. The simulation and projection of the land use pattern in the TGRA for the year 2030, as well as the anticipation of future trends of six types of ESs, were carried out. Compared with S Li, Q Xiong’s study [63, 64] on ecosystem services in the Three Gorges Reservoir area, the present study is relatively more comprehensive in terms of time span and results, and makes predictions about future ecosystem services. Furthermore, the study used global variable spatial autocorrelation and local variable spatial autocorrelation analysis methods for the simulated ESs types in 2030 to investigate the spatial correlation and aggregation characteristics between the future LULC pattern changes in the TGRA and various ESs. The study sought to analyse the mechanisms by which land use patterns affect ESs from the standpoints of politics, society, and technology.

However, there are certain limitations to this study. On the one hand, data resolution, such as land use, can affect the precision of ESs evaluations. Moreover, when modeling over long periods, the Geo SOS-FLUS model may encounter limits, thereby impacting forecast accuracy. The projection of grain production services, on the other hand, is based on existing development patterns mixed with predicted improvements in future agricultural production technology levels. The precision of this forecast is insufficient. Future studies will address the shortcomings identified above by investigating analytical methodologies more scientifically and accurately, notably in LULC prediction models and ESs evaluation methods. Furthermore, additional research into acquiring finer data will be undertaken in order to facilitate a more scientifically grounded study on land use simulation and ESs in the TGRA.

## 6. Conclusion

The TGRA is used as the research object in this work. We combine the FLUS and InVEST models, as well as the RUSLE model, using data such as land cover from 1990 to 2020, meteorological data, soil data, elevation data, NDVI data, and a GIS platform. The goal is to use the TGRA to model the future LULC pattern. In addition, the study intends to evaluate and forecast the efficacy of six types of ESs. From 1990 to 2020, the regional differences and temporal trends in six types of ecosystem services in the TGRA were studied. The study also looked at the influence of future changes in LULC patterns on ESs efficiency. The following are the particular primary conclusions:

1. From 1990 to 2020, the TGRA’s water conservation service values exhibited an initial fall followed by a rise. High-value areas are mostly located in the reservoir’s central and eastern sections, whereas low-value areas are mostly found in the western section. The soil retention services’ values initially declined and subsequently climbed. High-value areas are concentrated in Wuxi, Yiling, and Xingshan, whereas low-value areas are concentrated along the banks of the Yangtze River and in the central urban area. Carbon sequestration capacity has been rising year after year, with average carbon sequestration in 2020 being 11.45% more than in 2000, suggesting a considerable rise. The quality of habitat has remained generally steady, with only minor changes in spatial patterns, but a reduction was noted in 2020, indicating the possibility of ecological deterioration.
2. The following are the simulation findings for three LULC scenarios: Under the natural development scenario, carbon storage and soil retention achieve their maximum values while their organic matter production function declines. Under the natural development scenario, the carbon storage value in 2030 falls from 615.92 g/m^2^ in 2020 to 595.62 g/m^2^ in 2030. The development of residential land in Chongqing’s major metropolitan region has increased land area while decreasing carbon storage.
3. The four ecosystem services display positive geographical association, according to a global spatial autocorrelation study of the administrative divisions in the TGRA and the spatiotemporal variations in the four ESs under the three scenarios in 2030. Each ESs’s high and low-value areas are spatially adjacent, demonstrating a strong positive spatial correlation. Carbon storage, water yield, and habitat quality all show comparable regional distributions under the three scenarios, according to a local spatial autocorrelation study. High-value areas for ESs are found in the reservoir’s initial and middle parts, probably due to increased regional forest cover in these places.
4. Variations in ESs are caused by changes in the TGRA’s LULC pattern. Understanding the sustainable planning strategies for future LULC patterns is critical in the Yangtze River Basin, as an ecologically significant region in the Yangtze River Basin, a critical component of the Yangtze River Economic Belt, and a pivotal hub in the urban agglomerations of both the Middle and Upper Yangtze, as well as Chengdu-Chongqing. Exploring the harmonic route between high-level protection and high-quality development in this region is critical for the continuing growth of the Yangtze River Economic Belt’s high-quality development plan.

## References

[1] Bilotto F, Christie-Whitehead KM, Malcolm B, Harrison MT. Carbon, cash, cattle and the climate crisis. Sustain Sci. 2023;18: 1795–1811. doi: 10.1007/s11625-023-01323-2

[2] Lombardi D, Uslu B, Bailey JM. Extreme Weather Events and the Climate Crisis: What is the Connection? 2020.

[3] Su F, Liu Y, Chen S-J, Fahad S. Towards the impact of economic policy uncertainty on food security: Introducing a comprehensive heterogeneous framework for assessment. Journal of Cleaner Production. 2023;386: 135792. doi: 10.1016/j.jclepro.2022.135792

[4] Gonzalez A, Chase JM, O’Connor MI. A framework for the detection and attribution of biodiversity change. Philosophical Transactions of the Royal Society B: Biological Sciences. 2023;378: 20220182. doi: 10.1098/rstb.2022.0182 37246383 PMC10225858

[5] Panwar R, Ober H, Pinkse J. The uncomfortable relationship between business and biodiversity: Advancing research on business strategies for biodiversity protection. Business Strategy and the Environment. 2023;32: 2554–2566. doi: 10.1002/bse.3139

[6] Wei B, Peng Y, Lin L, Zhang D, Ma L, Jiang L, et al. Drivers of biochar-mediated improvement of soil water retention capacity based on soil texture: A meta-analysis. Geoderma. 2023;437: 116591. doi: 10.1016/j.geoderma.2023.116591

[7] Wanzek T, Stults JF, Johnson MG, Field JA, Kleber M. Role of Mineral–Organic Interactions in PFAS Retention by AFFF-Impacted Soil. Environ Sci Technol. 2023;57: 5231–5242. doi: 10.1021/acs.est.2c08806 36947878 PMC10764056

[8] Woon KS, Phuang ZX, Taler J, Varbanov PS, Chong CT, Klemeš JJ, et al. Recent advances in urban green energy development towards carbon emissions neutrality. Energy. 2023;267: 126502. doi: 10.1016/j.energy.2022.126502

[9] Zhou K, Yang J, Yang T, Ding T. Spatial and temporal evolution characteristics and spillover effects of China’s regional carbon emissions. Journal of Environmental Management. 2023;325: 116423. doi: 10.1016/j.jenvman.2022.116423 36244288

[10] Ledger SEH, Loh J, Almond R, Böhm M, Clements CF, Currie J, et al. Past, present, and future of the Living Planet Index. npj biodivers. 2023;2: 1–13. doi: 10.1038/s44185-023-00017-3 PMC11332142 39242663

[11] Gnacadja L, Vidal A. How can science help to implement the UN Decade on Ecosystem Restoration 2021–2030? Philosophical Transactions of the Royal Society B: Biological Sciences. 2022;378: 20210066. doi: 10.1098/rstb.2021.0066 36373916 PMC9661945

[12] Sivadas D. Pathways for Sustainable Economic Benefits and Green Economies in Light of the State of World Forests 2022. Anthr Sci. 2022;1: 460–465. doi: 10.1007/s44177-022-00041-1

[13] Hua F, Bruijnzeel LA, Meli P, Martin PA, Zhang J, Nakagawa S, et al. The biodiversity and ecosystem service contributions and trade-offs of forest restoration approaches. Science. 2022;376: 839–844. doi: 10.1126/science.abl4649 35298279

[14] Luck GW, Daily GC, Ehrlich PR. Population diversity and ecosystem services. Trends in Ecology & Evolution. 2003;18: 331–336. doi: 10.1016/S0169-5347(03)00100-9

[15] Zhang C, Li J, Zhou Z. Ecosystem service cascade: Concept, review, application and prospect. Ecological Indicators. 2022;137: 108766. doi: 10.1016/j.ecolind.2022.108766

[16] Balmford A, Bruner A, Cooper P, Costanza R, Farber S, Green RE, et al. Economic Reasons for Conserving Wild Nature. Science. 2002 [cited 29 Dec 2023]. doi: 10.1126/science.1073947 12169718

[17] Goehring DM, Daily GC, Şekerçioglu ÇH. Distribution of Ground-dwelling Arthropods in Tropical Countryside Habitats. Journal of Insect Conservation. 2002;6: 83–91. doi: 10.1023/A:1020905307244

[18] Lee H, Lautenbach S. A quantitative review of relationships between ecosystem services. Ecological Indicators. 2016;66: 340–351. doi: 10.1016/j.ecolind.2016.02.004

[19] Czúcz B, Arany I, Potschin-Young M, Bereczki K, Kertész M, Kiss M, et al. Where concepts meet the real world: A systematic review of ecosystem service indicators and their classification using CICES. Ecosystem Services. 2018;29: 145–157. doi: 10.1016/j.ecoser.2017.11.018

[20] Kopperoinen L, Itkonen P, Niemelä J. Using expert knowledge in combining green infrastructure and ecosystem services in land use planning: an insight into a new place-based methodology. Landscape Ecol. 2014;29: 1361–1375. doi: 10.1007/s10980-014-0014-2

[21] Rugani B, Maia de Souza D, Weidema BP, Bare J, Bakshi B, Grann B, et al. Towards integrating the ecosystem services cascade framework within the Life Cycle Assessment (LCA) cause-effect methodology. Science of The Total Environment. 2019;690: 1284–1298. doi: 10.1016/j.scitotenv.2019.07.023 31470491 PMC7791572

[22] Powledge F. The Millennium Assessment. BioScience. 2006;56: 880–886. doi: 10.1641/0006-3568(2006)56[880:TMA]2.0.CO;2

[23] Wu W, Zeng H, Guo C, You W, Xu H, Hu Y, et al. Spatial heterogeneity and management challenges of ecosystem service trade-offs: a case study in Guangdong Province, China. Environmental Management. 2023 [cited 29 Dec 2023]. doi: 10.1007/s00267-023-01851-8 37365302

[24] Padilha J, Carvalho-Santos C, Cássio F, Pascoal C. Land Cover Implications on Ecosystem Service Delivery: a Multi-Scenario Study of Trade-offs and Synergies in River Basins. Environmental Management. 2023 [cited 29 Dec 2023]. doi: 10.1007/s00267-023-01916-8 38063877

[25] Chen W, Chi G. Ecosystem services trade-offs and synergies in China, 2000–2015. Int J Environ Sci Technol. 2023;20: 3221–3236. doi: 10.1007/s13762-022-04141-8

[26] Xie L, Wang H, Liu S. The ecosystem service values simulation and driving force analysis based on land use/land cover: A case study in inland rivers in arid areas of the Aksu River Basin, China. Ecological Indicators. 2022;138: 108828. doi: 10.1016/j.ecolind.2022.108828

[27] Li Y, Liu W, Feng Q, Zhu M, Yang L, Zhang J. Quantitative Assessment for the Spatiotemporal Changes of Ecosystem Services, Tradeoff–Synergy Relationships and Drivers in the Semi-Arid Regions of China. Remote Sensing. 2022;14: 239. doi: 10.3390/rs14010239

[28] Zhang L, Fang C, Zhu C, Gao Q. Ecosystem service trade-offs and identification of eco-optimal regions in urban agglomerations in arid regions of China. Journal of Cleaner Production. 2022;373: 133823. doi: 10.1016/j.jclepro.2022.133823

[29] Qiu M, Wang Y, Sun C, Gao X. Dry-wet cycling area enhances soil ecosystem multifunctionality in the aquatic-terrestrial ecotones of the Caohai Lake in China. Environ Sci Pollut Res. 2023;30: 116363–116375. doi: 10.1007/s11356-023-30637-y 37910349

[30] Tavakoli M, Mohammadyari F. Modeling the spatial distribution of multiple ecosystem services in Ilam dam watershed, Western Iran: identification of areas for spatial planning. Urban Ecosyst. 2023;26: 459–478. doi: 10.1007/s11252-022-01297-6

[31] Tang Z, Zhou Z, Wang D, Luo F, Bai J, Fu Y. Impact of vegetation restoration on ecosystem services in the Loess plateau, a case study in the Jinghe Watershed, China. Ecological Indicators. 2022;142: 109183. doi: 10.1016/j.ecolind.2022.109183

[32] Inácio M, Gomes E, Bogdzevič K, Kalinauskas M, Zhao W, Pereira P. Mapping and assessing coastal recreation cultural ecosystem services supply, flow, and demand in Lithuania. Journal of Environmental Management. 2022;323: 116175. doi: 10.1016/j.jenvman.2022.116175 36088764

[33] Wang P, Wang J, Zhang J, Ma X, Zhou L, Sun Y. Spatial-temporal changes in ecosystem services and social-ecological drivers in a typical coastal tourism city: A case study of Sanya, China. Ecological Indicators. 2022;145: 109607. doi: 10.1016/j.ecolind.2022.109607

[34] Wang S, Hu M, Wang Y, Xia B. Dynamics of ecosystem services in response to urbanization across temporal and spatial scales in a mega metropolitan area. Sustainable Cities and Society. 2022;77: 103561. doi: 10.1016/j.scs.2021.103561

[35] Zhang D, Jing P, Sun P, Ren H, Ai Z. The non-significant correlation between landscape ecological risk and ecosystem services in Xi’an Metropolitan Area, China. Ecological Indicators. 2022;141: 109118. doi: 10.1016/j.ecolind.2022.109118

[36] Fang Z, Ding T, Chen J, Xue S, Zhou Q, Wang Y, et al. Impacts of land use/land cover changes on ecosystem services in ecologically fragile regions. Science of The Total Environment. 2022;831: 154967. doi: 10.1016/j.scitotenv.2022.154967 35367552

[37] Chen J, Wang S, Zou Y. Construction of an ecological security pattern based on ecosystem sensitivity and the importance of ecological services: A case study of the Guanzhong Plain urban agglomeration, China. Ecological Indicators. 2022;136: 108688. doi: 10.1016/j.ecolind.2022.108688

[38] Luiza Petroni M, Siqueira-Gay J, Lucia Casteli Figueiredo Gallardo A. Understanding land use change impacts on ecosystem services within urban protected areas. Landscape and Urban Planning. 2022;223: 104404. doi: 10.1016/j.landurbplan.2022.104404

[39] Ge G, Zhang J, Chen X, Liu X, Hao Y, Yang X, et al. Effects of land use and land cover change on ecosystem services in an arid desert-oasis ecotone along the Yellow River of China. Ecological Engineering. 2022;176: 106512. doi: 10.1016/j.ecoleng.2021.106512

[40] Wang P, Li R, Liu D, Wu Y. Dynamic characteristics and responses of ecosystem services under land use/land cover change scenarios in the Huangshui River Basin, China. Ecological Indicators. 2022;144: 109539. doi: 10.1016/j.ecolind.2022.109539

[41] Su Y, Feng Q, Liu W, Zhu M, Xia H, Ma X, et al. Improved Understanding of Trade-Offs and Synergies in Ecosystem Services via Fine Land-Use Classification and Multi-Scale Analysis in the Arid Region of Northwest China. Remote Sensing. 2023;15: 4976. doi: 10.3390/rs15204976

[42] Luo R, Yang S, Wang Z, Zhang T, Gao P. Impact and trade off analysis of land use change on spatial pattern of ecosystem services in Chishui River Basin. Environ Sci Pollut Res. 2022;29: 20234–20248. doi: 10.1007/s11356-021-17188-w 34729715

[43] Aksoy H, Kaptan S. Simulation of future forest and land use/cover changes (2019–2039) using the cellular automata-Markov model. Geocarto International. 2022;37: 1183–1202. doi: 10.1080/10106049.2020.1778102

[44] Liao G, He P, Gao X, Lin Z, Huang C, Zhou W, et al. Land use optimization of rural production–living–ecological space at different scales based on the BP–ANN and CLUE–S models. Ecological Indicators. 2022;137: 108710. doi: 10.1016/j.ecolind.2022.108710

[45] Liu X, Liang X, Li X, Xu X, Ou J, Chen Y, et al. A future land use simulation model (FLUS) for simulating multiple land use scenarios by coupling human and natural effects. Landscape and Urban Planning. 2017;168: 94–116. doi: 10.1016/j.landurbplan.2017.09.019

[46] Liu Y, Jing Y, Han S. Multi-scenario simulation of land use/land cover change and water yield evaluation coupled with the GMOP-PLUS-InVEST model: A case study of the Nansi Lake Basin in China. Ecological Indicators. 2023;155: 110926. doi: 10.1016/j.ecolind.2023.110926

[47] Qiao X, Li Z, Lin J, Wang H, Zheng S, Yang S. Assessing current and future soil erosion under changing land use based on InVEST and FLUS models in the Yihe River Basin, North China. International Soil and Water Conservation Research. 2023 [cited 29 Dec 2023]. doi: 10.1016/j.iswcr.2023.07.001

[48] Patterson TM, Coelho DL. Ecosystem services: Foundations, opportunities, and challenges for the forest products sector. Forest Ecology and Management. 2009;257: 1637–1646. doi: 10.1016/j.foreco.2008.11.010

[49] Costanza R, de Groot R, Sutton P, van der Ploeg S, Anderson SJ, Kubiszewski I, et al. Changes in the global value of ecosystem services. Global Environmental Change. 2014;26: 152–158. doi: 10.1016/j.gloenvcha.2014.04.002

[50] Li G, Cheng G, Wu Z. Resilience Assessment of Urban Complex Giant Systems in Hubei Section of the Three Gorges Reservoir Area Based on Multi-Source Data. Sustainability. 2022;14: 8423. doi: 10.3390/su14148423

[51] Li G, Cheng G, Wu Z, Liu X. Coupling Coordination Research on Disaster-Adapted Resilience of Modern Infrastructure System in the Middle and Lower Section of the Three Gorges Reservoir Area. Sustainability. 2022;14: 14514. doi: 10.3390/su142114514

[52] Tan Z, Guan Q, Lin J, Yang L, Luo H, Ma Y, et al. The response and simulation of ecosystem services value to land use/land cover in an oasis, Northwest China. Ecological Indicators. 2020;118: 106711. doi: 10.1016/j.ecolind.2020.106711

[53] Mohammadyari F, Zarandian A, Mirsanjari MM, Suziedelyte Visockiene J, Tumeliene E. Modelling Impact of Urban Expansion on Ecosystem Services: A Scenario-Based Approach in a Mixed Natural/Urbanised Landscape. Land. 2023;12: 291. doi: 10.3390/land12020291

[54] Neumann K, Verburg PH, Stehfest E, Müller C. The yield gap of global grain production: A spatial analysis. Agricultural Systems. 2010;103: 316–326. doi: 10.1016/j.agsy.2010.02.004

[55] Redhead JW, Stratford C, Sharps K, Jones L, Ziv G, Clarke D, et al. Empirical validation of the InVEST water yield ecosystem service model at a national scale. Science of The Total Environment. 2016;569–570: 1418–1426. doi: 10.1016/j.scitotenv.2016.06.227 27395076

[56] Babbar D, Areendran G, Sahana M, Sarma K, Raj K, Sivadas A. Assessment and prediction of carbon sequestration using Markov chain and InVEST model in Sariska Tiger Reserve, India. Journal of Cleaner Production. 2021;278: 123333. doi: 10.1016/j.jclepro.2020.123333

[57] Mondal A, Khare D, Kundu S. A comparative study of soil erosion modelling by MMF, USLE and RUSLE. Geocarto International. 2018;33: 89–103. doi: 10.1080/10106049.2016.1232313

[58] Wu L, Sun C, Fan F. Estimating the Characteristic Spatiotemporal Variation in Habitat Quality Using the InVEST Model—A Case Study from Guangdong–Hong Kong–Macao Greater Bay Area. Remote Sensing. 2021;13: 1008. doi: 10.3390/rs13051008

[59] Yuan J, Niu Z, Wang C. Vegetation NPP distribution based on MODIS data and CASA model—A case study of northern Hebei Province. Chin GeographSc. 2006;16: 334–341. doi: 10.1007/s11769-006-0334-5

[60] Dormann CF. Effects of incorporating spatial autocorrelation into the analysis of species distribution data. Global Ecology and Biogeography. 2007;16: 129–138. doi: 10.1111/j.1466-8238.2006.00279.x

[61] Shaikh SFEA See SC, Richards D Belcher RN, Grêt-Regamey A, Galleguillos Torres M, et al. Accounting for spatial autocorrelation is needed to avoid misidentifying trade-offs and bundles among ecosystem services. Ecological Indicators. 2021;129: 107992. doi: 10.1016/j.ecolind.2021.107992

[62] Zhang Y, Liu Y, Zhang Y, Liu Y, Zhang G, Chen Y. On the spatial relationship between ecosystem services and urbanization: A case study in Wuhan, China. Science of The Total Environment. 2018;637–638: 780–790. doi: 10.1016/j.scitotenv.2018.04.396 29758433

[63] Li S, Bing Z, Jin G. Spatially Explicit Mapping of Soil Conservation Service in Monetary Units Due to Land Use/Cover Change for the Three Gorges Reservoir Area, China. Remote Sensing. 2019;11: 468. doi: 10.3390/rs11040468

[64] Xiong Q, Xiao Y, Halmy MWA, Pan K, Dakhil MA, Zhang L, et al. A blessing for the Yangtze River: optimization of Chinese regional policy planning for water yield and purification in the Three Gorges Reservoir Area. Environ Sci Pollut Res. 2020;27: 7040–7052. doi: 10.1007/s11356-019-07178-4 31883073

